# Supplementary material for: Incorporating wellbeing into general factor models: A more complete mental state?
Source: PLoS One. 2025 Nov 17;20(11):e0335657. doi: 10.1371/journal.pone.0335657 (PMC12622774; doi:10.1371/journal.pone.0335657)
Supplement: S1 Table — (DOCX) [file pone.0335657.s001.docx]

|  | **Sample who were followed up (N=11535)** | **Sample who were not followed up (N=3723)** | **Statistical Test** |
| --- | --- | --- | --- |
| Gender | F: 6221 (53.9%)  M: 5314 (46.1%)  Missing: 0* | F: 1895 (51.5%)  M: 1788 (48.5%)  Missing: 40 | X-squared = 6.89, p-value < 0.001  Significantly more proportion of males not followed up |
| Ethnicity | Asian: 1205 (10.7%)  Black: 701 (6.2%)  Mixed: 471 (4.2%)  Other: 345 (3.1%)  White: 8578 (75.9%)  Missing: 235 | Asian: 264 (7.7%)  Black: 171 (5.0%)  Mixed: 144 (4.2%)  Other: 142 (4.1%)  White: 2716 (79.0%)  Missing: 286 | X-squared = 42.844, p-value < 0.001  Significantly more proportion of White children not followed up as compared to Asian, Black and Other ethnic groups |
| Free School Meals | No: 7404 (65.5%)  Yes: 3896 (34.5%)  Missing: 235 | No: 1962 (57.1%)  Yes: 1475 (42.9%)  Missing: 286 | X-squared = 81.0, p-value < 0.001  Significantly more proportion of those eligible for FSM were not followed up |
| SEN Provision | No: 9943 (88.8%)  Yes: 1250 (11.2%)  Missing: 342 | No: 2833 (84.3%)  Yes: 527 (15.7%)  Missing: 363 | X-squared = 49.2, p-value = 2.33e-12  Significantly more proportion of those with SEN provision were not followed up |
| IDACI score | 0.246 | 0.254 | T = -2.92, p <0.01  Those living in more deprived areas more likely to be not followed up |
| SDQ Emotional Score at baseline | 3.80 | 3.98 | t = -3.81, p < 0.001  Those with higher emotional problems score at dp1 were more likely to be not followed up |
| SDQ Conduct Score at baseline | 2.40 | 2.82 | t = -10.61, p = 2.2e-16  Those with higher emotional problems score at dp1 were more likely to be not followed up |
| SWEMWEBS score at baseline | 24.6 | 23.9 | t = 6.5, p = 8.8e-11  Those with lesser wellbeing at dp1 were more likely to be not followed up |
| Impact score at baseline | 1.95 | 2.37 | t = -7.7 , p = 2.13e-14  Those with higher impairment at dp1 were more likely to be not followed up |

**S1 Table. Missingness by variables of interest at baseline.**

*Note that missing values in predictors were not included in statistical tests.
