# Supplementary material for: Incorporating wellbeing into general factor models: A more complete mental state?
Source: PLoS One. 2025 Nov 17;20(11):e0335657. doi: 10.1371/journal.pone.0335657 (PMC12622774; doi:10.1371/journal.pone.0335657)
Supplement: S2 Table — (DOCX) [file pone.0335657.s002.docx]

**S2 Table. Results of multiple imputation for variables of interest.**

| **Variable** | **Complete cases (missing values excluded per variable)** | **Imputed datasets (m=20)*** |
| --- | --- | --- |
| SDQ Item 1_t1 | Mean: 1.62  NA: 83 | Mean: 1.62 |
| SWEMWBS Item 1_t1 | Mean: 3.35  NA: 252 | Mean: 3.35 |
| Impact score_t1 | Mean: 2.05  NA: 884 | Mean: 2.08 |
| Impact score_t2 | Mean: 2.38  NA: 4347 | Mean: 2.45 |
| Gender | F: 8116​  M: 7102​  NA: 40 | F: 8132  M: 7126 |
| Ethnicity | 1: 1469  2: 872​  3: 615  4: 487​  5: 11294  NA: 521 | 1: 1531  2: 915​  3: 646  4: 516​  5: 11650 |
| IDACI Score | Mean: 0.25​  NA: 541 | Mean: 0.25 |
| FSM status | 0: 9366​  1: 5371​  NA: 521 | 0: 9685​  1: 5573 |
| SEN status | 0: 12776​  1: 1777​  NA: 705 | 0: 13333​  1: 1925 |

*Both means and frequencies reported are of the first imputed dataset. Other imputed datasets had similar means and frequencies.

**Intepretation:** Imputation diagnostics suggest that the imputed data have plausible values which are close to the complete case dataset and as expected, the imputed datasets have slightly higher means for the impact score variable, to account for the finding that children with higher impact scores were more likely to have missing data.
