# Supplementary material for: Incorporating wellbeing into general factor models: A more complete mental state?
Source: PLoS One. 2025 Nov 17;20(11):e0335657. doi: 10.1371/journal.pone.0335657 (PMC12622774; doi:10.1371/journal.pone.0335657)
Supplement: S3 Table — (DOCX) [file pone.0335657.s003.docx]

**S3 Table. Factor loadings for measurement models.**

**S3 Table A.** Factor loadings for bifactor g_wb_ model

| Variable | Estimate | Standard Error | P value | Standardised Estimate |
| --- | --- | --- | --- | --- |
| Int =~ dp1_sdq3 | 1.00 | 0.00 | NA | 0.25 |
| Int =~ dp1_sdq8 | 2.53 | 0.12 | <0.001 | 0.64 |
| Int =~ dp1_sdq13 | 1.48 | 0.07 | <0.001 | 0.38 |
| Int =~ dp1_sdq16 | 2.11 | 0.11 | <0.001 | 0.53 |
| Int =~ dp1_sdq24 | 2.42 | 0.12 | <0.001 | 0.61 |
| Ext =~ dp1_sdq5 | 1.00 | 0.00 | NA | 0.47 |
| Ext =~ dp1_sdq7_reversed | 0.91 | 0.03 | <0.001 | 0.42 |
| Ext =~ dp1_sdq12 | 1.65 | 0.05 | <0.001 | 0.77 |
| Ext =~ dp1_sdq18 | 0.88 | 0.03 | <0.001 | 0.41 |
| Ext =~ dp1_sdq22 | 1.03 | 0.04 | <0.001 | 0.48 |
| WB =~ dp1_swemwebs1 | 1.00 | 0.00 | NA | 0.45 |
| WB =~ dp1_swemwebs2 | 1.17 | 0.03 | <0.001 | 0.53 |
| WB =~ dp1_swemwebs3 | 0.75 | 0.03 | <0.001 | 0.34 |
| WB =~ dp1_swemwebs4 | 0.87 | 0.03 | <0.001 | 0.40 |
| WB =~ dp1_swemwebs5 | 0.97 | 0.03 | <0.001 | 0.44 |
| WB =~ dp1_swemwebs6 | 1.04 | 0.03 | <0.001 | 0.47 |
| WB =~ dp1_swemwebs7 | 0.97 | 0.03 | <0.001 | 0.44 |
| c =~ dp1_sdq3 | 1.00 | 0.00 | NA | 0.44 |
| c =~ dp1_sdq8 | 1.00 | 0.03 | <0.001 | 0.44 |
| c =~ dp1_sdq13 | 1.55 | 0.04 | <0.001 | 0.68 |
| c =~ dp1_sdq16 | 0.85 | 0.03 | <0.001 | 0.37 |
| c =~ dp1_sdq24 | 0.76 | 0.03 | <0.001 | 0.33 |
| c =~ dp1_sdq5 | 1.23 | 0.04 | <0.001 | 0.54 |
| c =~ dp1_sdq7_reversed | 0.77 | 0.03 | <0.001 | 0.34 |
| c =~ dp1_sdq12 | 0.75 | 0.03 | <0.001 | 0.33 |
| c =~ dp1_sdq18 | 1.06 | 0.03 | <0.001 | 0.46 |
| c =~ dp1_sdq22 | 0.65 | 0.03 | <0.001 | 0.29 |
| c =~ dp1_swemwebs1 | -0.63 | 0.03 | <0.001 | -0.27 |
| c =~ dp1_swemwebs2 | -0.93 | 0.03 | <0.001 | -0.41 |
| c =~ dp1_swemwebs3 | -1.26 | 0.04 | <0.001 | -0.55 |
| c =~ dp1_swemwebs4 | -1.27 | 0.04 | <0.001 | -0.56 |
| c =~ dp1_swemwebs5 | -1.41 | 0.04 | <0.001 | -0.62 |
| c =~ dp1_swemwebs6 | -0.74 | 0.03 | <0.001 | -0.32 |
| c =~ dp1_swemwebs7 | -1.16 | 0.04 | <0.001 | -0.51 |
| dp1_sdq3 \| t1 | -0.17 | 0.01 | <0.001 | -0.17 |
| dp1_sdq3 \| t2 | 0.95 | 0.01 | <0.001 | 0.95 |
| dp1_sdq8 \| t1 | -0.44 | 0.01 | <0.001 | -0.44 |
| dp1_sdq8 \| t2 | 0.62 | 0.01 | <0.001 | 0.62 |
| dp1_sdq13 \| t1 | 0.19 | 0.01 | <0.001 | 0.19 |
| dp1_sdq13 \| t2 | 1.24 | 0.01 | <0.001 | 1.24 |
| dp1_sdq16 \| t1 | -0.54 | 0.01 | <0.001 | -0.54 |
| dp1_sdq16 \| t2 | 0.58 | 0.01 | <0.001 | 0.58 |
| dp1_sdq24 \| t1 | 0.00 | 0.01 | 0.669 | 0.00 |
| dp1_sdq24 \| t2 | 1.05 | 0.01 | <0.001 | 1.05 |
| dp1_sdq5 \| t1 | -0.23 | 0.01 | <0.001 | -0.23 |
| dp1_sdq5 \| t2 | 0.77 | 0.01 | <0.001 | 0.77 |
| dp1_sdq7_reversed \| t1 | -0.14 | 0.01 | <0.001 | -0.14 |
| dp1_sdq7_reversed \| t2 | 1.54 | 0.02 | <0.001 | 1.54 |
| dp1_sdq12 \| t1 | 0.71 | 0.01 | <0.001 | 0.71 |
| dp1_sdq12 \| t2 | 1.66 | 0.02 | <0.001 | 1.66 |
| dp1_sdq18 \| t1 | 0.14 | 0.01 | <0.001 | 0.14 |
| dp1_sdq18 \| t2 | 1.05 | 0.01 | <0.001 | 1.05 |
| dp1_sdq22 \| t1 | 1.02 | 0.01 | <0.001 | 1.02 |
| dp1_sdq22 \| t2 | 1.77 | 0.02 | <0.001 | 1.77 |
| dp1_swemwebs1 \| t1 | -1.47 | 0.02 | <0.001 | -1.47 |
| dp1_swemwebs1 \| t2 | -0.78 | 0.01 | <0.001 | -0.78 |
| dp1_swemwebs1 \| t3 | 0.07 | 0.01 | <0.001 | 0.07 |
| dp1_swemwebs1 \| t4 | 0.99 | 0.01 | <0.001 | 0.99 |
| dp1_swemwebs2 \| t1 | -1.52 | 0.02 | <0.001 | -1.52 |
| dp1_swemwebs2 \| t2 | -0.74 | 0.01 | <0.001 | -0.74 |
| dp1_swemwebs2 \| t3 | 0.17 | 0.01 | <0.001 | 0.17 |
| dp1_swemwebs2 \| t4 | 1.17 | 0.01 | <0.001 | 1.17 |
| dp1_swemwebs3 \| t1 | -1.53 | 0.02 | <0.001 | -1.53 |
| dp1_swemwebs3 \| t2 | -0.68 | 0.01 | <0.001 | -0.68 |
| dp1_swemwebs3 \| t3 | 0.16 | 0.01 | <0.001 | 0.16 |
| dp1_swemwebs3 \| t4 | 1.02 | 0.01 | <0.001 | 1.02 |
| dp1_swemwebs4 \| t1 | -1.45 | 0.02 | <0.001 | -1.45 |
| dp1_swemwebs4 \| t2 | -0.75 | 0.01 | <0.001 | -0.75 |
| dp1_swemwebs4 \| t3 | 0.05 | 0.01 | <0.001 | 0.05 |
| dp1_swemwebs4 \| t4 | 0.92 | 0.01 | <0.001 | 0.92 |
| dp1_swemwebs5 \| t1 | -1.71 | 0.02 | <0.001 | -1.71 |
| dp1_swemwebs5 \| t2 | -0.96 | 0.01 | <0.001 | -0.96 |
| dp1_swemwebs5 \| t3 | -0.10 | 0.01 | <0.001 | -0.10 |
| dp1_swemwebs5 \| t4 | 0.83 | 0.01 | <0.001 | 0.83 |
| dp1_swemwebs6 \| t1 | -1.63 | 0.02 | <0.001 | -1.63 |
| dp1_swemwebs6 \| t2 | -0.97 | 0.01 | <0.001 | -0.97 |
| dp1_swemwebs6 \| t3 | -0.26 | 0.01 | <0.001 | -0.26 |
| dp1_swemwebs6 \| t4 | 0.57 | 0.01 | <0.001 | 0.57 |
| dp1_swemwebs7 \| t1 | -1.91 | 0.02 | <0.001 | -1.91 |
| dp1_swemwebs7 \| t2 | -1.27 | 0.01 | <0.001 | -1.27 |
| dp1_swemwebs7 \| t3 | -0.49 | 0.01 | <0.001 | -0.49 |
| dp1_swemwebs7 \| t4 | 0.40 | 0.01 | <0.001 | 0.40 |
| dp1_sdq3 ~~ dp1_sdq3 | 0.74 | 0.00 | NA | 0.74 |
| dp1_sdq8 ~~ dp1_sdq8 | 0.40 | 0.00 | NA | 0.40 |
| dp1_sdq13 ~~ dp1_sdq13 | 0.40 | 0.00 | NA | 0.40 |
| dp1_sdq16 ~~ dp1_sdq16 | 0.58 | 0.00 | NA | 0.58 |
| dp1_sdq24 ~~ dp1_sdq24 | 0.51 | 0.00 | NA | 0.51 |
| dp1_sdq5 ~~ dp1_sdq5 | 0.49 | 0.00 | NA | 0.49 |
| dp1_sdq7_reversed ~~ dp1_sdq7_reversed | 0.71 | 0.00 | NA | 0.71 |
| dp1_sdq12 ~~ dp1_sdq12 | 0.30 | 0.00 | NA | 0.30 |
| dp1_sdq18 ~~ dp1_sdq18 | 0.62 | 0.00 | NA | 0.62 |
| dp1_sdq22 ~~ dp1_sdq22 | 0.69 | 0.00 | NA | 0.69 |
| dp1_swemwebs1 ~~ dp1_swemwebs1 | 0.72 | 0.00 | NA | 0.72 |
| dp1_swemwebs2 ~~ dp1_swemwebs2 | 0.56 | 0.00 | NA | 0.56 |
| dp1_swemwebs3 ~~ dp1_swemwebs3 | 0.58 | 0.00 | NA | 0.58 |
| dp1_swemwebs4 ~~ dp1_swemwebs4 | 0.53 | 0.00 | NA | 0.53 |
| dp1_swemwebs5 ~~ dp1_swemwebs5 | 0.42 | 0.00 | NA | 0.42 |
| dp1_swemwebs6 ~~ dp1_swemwebs6 | 0.67 | 0.00 | NA | 0.67 |
| dp1_swemwebs7 ~~ dp1_swemwebs7 | 0.55 | 0.00 | NA | 0.55 |
| Int ~~ Int | 0.06 | 0.01 | <0.001 | 1.00 |
| Ext ~~ Ext | 0.22 | 0.01 | <0.001 | 1.00 |
| WB ~~ WB | 0.21 | 0.01 | <0.001 | 1.00 |
| c ~~ c | 0.19 | 0.01 | <0.001 | 1.00 |
| Int ~~ Ext | 0.00 | 0.00 | NA | 0.00 |
| Int ~~ WB | 0.00 | 0.00 | NA | 0.00 |
| Int ~~ c | 0.00 | 0.00 | NA | 0.00 |
| Ext ~~ WB | 0.00 | 0.00 | NA | 0.00 |
| Ext ~~ c | 0.00 | 0.00 | NA | 0.00 |
| WB ~~ c | 0.00 | 0.00 | NA | 0.00 |
| dp1_sdq3 ~*~ dp1_sdq3 | 1.00 | 0.00 | NA | 1.00 |
| dp1_sdq8 ~*~ dp1_sdq8 | 1.00 | 0.00 | NA | 1.00 |
| dp1_sdq13 ~*~ dp1_sdq13 | 1.00 | 0.00 | NA | 1.00 |
| dp1_sdq16 ~*~ dp1_sdq16 | 1.00 | 0.00 | NA | 1.00 |
| dp1_sdq24 ~*~ dp1_sdq24 | 1.00 | 0.00 | NA | 1.00 |
| dp1_sdq5 ~*~ dp1_sdq5 | 1.00 | 0.00 | NA | 1.00 |
| dp1_sdq7_reversed ~*~ dp1_sdq7_reversed | 1.00 | 0.00 | NA | 1.00 |
| dp1_sdq12 ~*~ dp1_sdq12 | 1.00 | 0.00 | NA | 1.00 |
| dp1_sdq18 ~*~ dp1_sdq18 | 1.00 | 0.00 | NA | 1.00 |
| dp1_sdq22 ~*~ dp1_sdq22 | 1.00 | 0.00 | NA | 1.00 |
| dp1_swemwebs1 ~*~ dp1_swemwebs1 | 1.00 | 0.00 | NA | 1.00 |
| dp1_swemwebs2 ~*~ dp1_swemwebs2 | 1.00 | 0.00 | NA | 1.00 |
| dp1_swemwebs3 ~*~ dp1_swemwebs3 | 1.00 | 0.00 | NA | 1.00 |
| dp1_swemwebs4 ~*~ dp1_swemwebs4 | 1.00 | 0.00 | NA | 1.00 |
| dp1_swemwebs5 ~*~ dp1_swemwebs5 | 1.00 | 0.00 | NA | 1.00 |
| dp1_swemwebs6 ~*~ dp1_swemwebs6 | 1.00 | 0.00 | NA | 1.00 |
| dp1_swemwebs7 ~*~ dp1_swemwebs7 | 1.00 | 0.00 | NA | 1.00 |
| dp1_sdq3 ~1 | 0.00 | 0.00 | NA | 0.00 |
| dp1_sdq8 ~1 | 0.00 | 0.00 | NA | 0.00 |
| dp1_sdq13 ~1 | 0.00 | 0.00 | NA | 0.00 |
| dp1_sdq16 ~1 | 0.00 | 0.00 | NA | 0.00 |
| dp1_sdq24 ~1 | 0.00 | 0.00 | NA | 0.00 |
| dp1_sdq5 ~1 | 0.00 | 0.00 | NA | 0.00 |
| dp1_sdq7_reversed ~1 | 0.00 | 0.00 | NA | 0.00 |
| dp1_sdq12 ~1 | 0.00 | 0.00 | NA | 0.00 |
| dp1_sdq18 ~1 | 0.00 | 0.00 | NA | 0.00 |
| dp1_sdq22 ~1 | 0.00 | 0.00 | NA | 0.00 |
| dp1_swemwebs1 ~1 | 0.00 | 0.00 | NA | 0.00 |
| dp1_swemwebs2 ~1 | 0.00 | 0.00 | NA | 0.00 |
| dp1_swemwebs3 ~1 | 0.00 | 0.00 | NA | 0.00 |
| dp1_swemwebs4 ~1 | 0.00 | 0.00 | NA | 0.00 |
| dp1_swemwebs5 ~1 | 0.00 | 0.00 | NA | 0.00 |
| dp1_swemwebs6 ~1 | 0.00 | 0.00 | NA | 0.00 |
| dp1_swemwebs7 ~1 | 0.00 | 0.00 | NA | 0.00 |
| Int ~1 | 0.00 | 0.00 | NA | 0.00 |
| Ext ~1 | 0.00 | 0.00 | NA | 0.00 |
| WB ~1 | 0.00 | 0.00 | NA | 0.00 |
| c ~1 | 0.00 | 0.00 | NA | 0.00 |

**S3 Table B.** Factor loadings for correlated factors model.

| Variable | Estimate | Sndard Error | P value | Standardised Estimate |
| --- | --- | --- | --- | --- |
| Int =~ dp1_sdq3 | 1.00 | 0.00 | NA | 0.54 |
| Int =~ dp1_sdq8 | 1.29 | 0.02 | <0.001 | 0.70 |
| Int =~ dp1_sdq13 | 1.58 | 0.03 | <0.001 | 0.85 |
| Int =~ dp1_sdq16 | 1.11 | 0.02 | <0.001 | 0.60 |
| Int =~ dp1_sdq24 | 1.11 | 0.02 | <0.001 | 0.60 |
| Ext =~ dp1_sdq5 | 1.00 | 0.00 | NA | 0.78 |
| Ext =~ dp1_sdq7_reversed | 0.68 | 0.01 | <0.001 | 0.53 |
| Ext =~ dp1_sdq12 | 0.83 | 0.02 | <0.001 | 0.65 |
| Ext =~ dp1_sdq18 | 0.85 | 0.02 | <0.001 | 0.66 |
| Ext =~ dp1_sdq22 | 0.65 | 0.02 | <0.001 | 0.51 |
| WB =~ dp1_swemwebs1 | 1.00 | 0.00 | NA | 0.48 |
| WB =~ dp1_swemwebs2 | 1.32 | 0.02 | <0.001 | 0.63 |
| WB =~ dp1_swemwebs3 | 1.38 | 0.02 | <0.001 | 0.66 |
| WB =~ dp1_swemwebs4 | 1.45 | 0.02 | <0.001 | 0.69 |
| WB =~ dp1_swemwebs5 | 1.62 | 0.03 | <0.001 | 0.77 |
| WB =~ dp1_swemwebs6 | 1.11 | 0.02 | <0.001 | 0.53 |
| WB =~ dp1_swemwebs7 | 1.41 | 0.02 | <0.001 | 0.67 |
| dp1_sdq3 \| t1 | -0.17 | 0.01 | <0.001 | -0.17 |
| dp1_sdq3 \| t2 | 0.95 | 0.01 | <0.001 | 0.95 |
| dp1_sdq8 \| t1 | -0.44 | 0.01 | <0.001 | -0.44 |
| dp1_sdq8 \| t2 | 0.62 | 0.01 | <0.001 | 0.62 |
| dp1_sdq13 \| t1 | 0.19 | 0.01 | <0.001 | 0.19 |
| dp1_sdq13 \| t2 | 1.24 | 0.01 | <0.001 | 1.24 |
| dp1_sdq16 \| t1 | -0.54 | 0.01 | <0.001 | -0.54 |
| dp1_sdq16 \| t2 | 0.58 | 0.01 | <0.001 | 0.58 |
| dp1_sdq24 \| t1 | 0.00 | 0.01 | 0.668 | 0.00 |
| dp1_sdq24 \| t2 | 1.05 | 0.01 | <0.001 | 1.05 |
| dp1_sdq5 \| t1 | -0.23 | 0.01 | <0.001 | -0.23 |
| dp1_sdq5 \| t2 | 0.77 | 0.01 | <0.001 | 0.77 |
| dp1_sdq7_reversed \| t1 | -0.14 | 0.01 | <0.001 | -0.14 |
| dp1_sdq7_reversed \| t2 | 1.54 | 0.02 | <0.001 | 1.54 |
| dp1_sdq12 \| t1 | 0.71 | 0.01 | <0.001 | 0.71 |
| dp1_sdq12 \| t2 | 1.66 | 0.02 | <0.001 | 1.66 |
| dp1_sdq18 \| t1 | 0.14 | 0.01 | <0.001 | 0.14 |
| dp1_sdq18 \| t2 | 1.05 | 0.01 | <0.001 | 1.05 |
| dp1_sdq22 \| t1 | 1.02 | 0.01 | <0.001 | 1.02 |
| dp1_sdq22 \| t2 | 1.77 | 0.02 | <0.001 | 1.77 |
| dp1_swemwebs1 \| t1 | -1.47 | 0.02 | <0.001 | -1.47 |
| dp1_swemwebs1 \| t2 | -0.78 | 0.01 | <0.001 | -0.78 |
| dp1_swemwebs1 \| t3 | 0.07 | 0.01 | <0.001 | 0.07 |
| dp1_swemwebs1 \| t4 | 0.99 | 0.01 | <0.001 | 0.99 |
| dp1_swemwebs2 \| t1 | -1.52 | 0.02 | <0.001 | -1.52 |
| dp1_swemwebs2 \| t2 | -0.74 | 0.01 | <0.001 | -0.74 |
| dp1_swemwebs2 \| t3 | 0.17 | 0.01 | <0.001 | 0.17 |
| dp1_swemwebs2 \| t4 | 1.17 | 0.01 | <0.001 | 1.17 |
| dp1_swemwebs3 \| t1 | -1.53 | 0.02 | <0.001 | -1.53 |
| dp1_swemwebs3 \| t2 | -0.68 | 0.01 | <0.001 | -0.68 |
| dp1_swemwebs3 \| t3 | 0.16 | 0.01 | <0.001 | 0.16 |
| dp1_swemwebs3 \| t4 | 1.02 | 0.01 | <0.001 | 1.02 |
| dp1_swemwebs4 \| t1 | -1.45 | 0.02 | <0.001 | -1.45 |
| dp1_swemwebs4 \| t2 | -0.75 | 0.01 | <0.001 | -0.75 |
| dp1_swemwebs4 \| t3 | 0.05 | 0.01 | <0.001 | 0.05 |
| dp1_swemwebs4 \| t4 | 0.92 | 0.01 | <0.001 | 0.92 |
| dp1_swemwebs5 \| t1 | -1.71 | 0.02 | <0.001 | -1.71 |
| dp1_swemwebs5 \| t2 | -0.96 | 0.01 | <0.001 | -0.96 |
| dp1_swemwebs5 \| t3 | -0.10 | 0.01 | <0.001 | -0.10 |
| dp1_swemwebs5 \| t4 | 0.83 | 0.01 | <0.001 | 0.83 |
| dp1_swemwebs6 \| t1 | -1.63 | 0.02 | <0.001 | -1.63 |
| dp1_swemwebs6 \| t2 | -0.97 | 0.01 | <0.001 | -0.97 |
| dp1_swemwebs6 \| t3 | -0.26 | 0.01 | <0.001 | -0.26 |
| dp1_swemwebs6 \| t4 | 0.57 | 0.01 | <0.001 | 0.57 |
| dp1_swemwebs7 \| t1 | -1.91 | 0.02 | <0.001 | -1.91 |
| dp1_swemwebs7 \| t2 | -1.27 | 0.01 | <0.001 | -1.27 |
| dp1_swemwebs7 \| t3 | -0.49 | 0.01 | <0.001 | -0.49 |
| dp1_swemwebs7 \| t4 | 0.40 | 0.01 | <0.001 | 0.40 |
| dp1_sdq3 ~~ dp1_sdq3 | 0.71 | 0.00 | NA | 0.71 |
| dp1_sdq8 ~~ dp1_sdq8 | 0.51 | 0.00 | NA | 0.51 |
| dp1_sdq13 ~~ dp1_sdq13 | 0.28 | 0.00 | NA | 0.28 |
| dp1_sdq16 ~~ dp1_sdq16 | 0.64 | 0.00 | NA | 0.64 |
| dp1_sdq24 ~~ dp1_sdq24 | 0.64 | 0.00 | NA | 0.64 |
| dp1_sdq5 ~~ dp1_sdq5 | 0.39 | 0.00 | NA | 0.39 |
| dp1_sdq7_reversed ~~ dp1_sdq7_reversed | 0.71 | 0.00 | NA | 0.71 |
| dp1_sdq12 ~~ dp1_sdq12 | 0.57 | 0.00 | NA | 0.57 |
| dp1_sdq18 ~~ dp1_sdq18 | 0.56 | 0.00 | NA | 0.56 |
| dp1_sdq22 ~~ dp1_sdq22 | 0.74 | 0.00 | NA | 0.74 |
| dp1_swemwebs1 ~~ dp1_swemwebs1 | 0.77 | 0.00 | NA | 0.77 |
| dp1_swemwebs2 ~~ dp1_swemwebs2 | 0.61 | 0.00 | NA | 0.61 |
| dp1_swemwebs3 ~~ dp1_swemwebs3 | 0.56 | 0.00 | NA | 0.56 |
| dp1_swemwebs4 ~~ dp1_swemwebs4 | 0.52 | 0.00 | NA | 0.52 |
| dp1_swemwebs5 ~~ dp1_swemwebs5 | 0.40 | 0.00 | NA | 0.40 |
| dp1_swemwebs6 ~~ dp1_swemwebs6 | 0.72 | 0.00 | NA | 0.72 |
| dp1_swemwebs7 ~~ dp1_swemwebs7 | 0.55 | 0.00 | NA | 0.55 |
| Int ~~ Int | 0.29 | 0.01 | <0.001 | 1.00 |
| Ext ~~ Ext | 0.61 | 0.01 | <0.001 | 1.00 |
| WB ~~ WB | 0.23 | 0.01 | <0.001 | 1.00 |
| Int ~~ Ext | 0.19 | 0.01 | <0.001 | 0.44 |
| Int ~~ WB | -0.13 | 0.00 | <0.001 | -0.52 |
| Ext ~~ WB | -0.18 | 0.00 | <0.001 | -0.48 |
| dp1_sdq3 ~*~ dp1_sdq3 | 1.00 | 0.00 | NA | 1.00 |
| dp1_sdq8 ~*~ dp1_sdq8 | 1.00 | 0.00 | NA | 1.00 |
| dp1_sdq13 ~*~ dp1_sdq13 | 1.00 | 0.00 | NA | 1.00 |
| dp1_sdq16 ~*~ dp1_sdq16 | 1.00 | 0.00 | NA | 1.00 |
| dp1_sdq24 ~*~ dp1_sdq24 | 1.00 | 0.00 | NA | 1.00 |
| dp1_sdq5 ~*~ dp1_sdq5 | 1.00 | 0.00 | NA | 1.00 |
| dp1_sdq7_reversed ~*~ dp1_sdq7_reversed | 1.00 | 0.00 | NA | 1.00 |
| dp1_sdq12 ~*~ dp1_sdq12 | 1.00 | 0.00 | NA | 1.00 |
| dp1_sdq18 ~*~ dp1_sdq18 | 1.00 | 0.00 | NA | 1.00 |
| dp1_sdq22 ~*~ dp1_sdq22 | 1.00 | 0.00 | NA | 1.00 |
| dp1_swemwebs1 ~*~ dp1_swemwebs1 | 1.00 | 0.00 | NA | 1.00 |
| dp1_swemwebs2 ~*~ dp1_swemwebs2 | 1.00 | 0.00 | NA | 1.00 |
| dp1_swemwebs3 ~*~ dp1_swemwebs3 | 1.00 | 0.00 | NA | 1.00 |
| dp1_swemwebs4 ~*~ dp1_swemwebs4 | 1.00 | 0.00 | NA | 1.00 |
| dp1_swemwebs5 ~*~ dp1_swemwebs5 | 1.00 | 0.00 | NA | 1.00 |
| dp1_swemwebs6 ~*~ dp1_swemwebs6 | 1.00 | 0.00 | NA | 1.00 |
| dp1_swemwebs7 ~*~ dp1_swemwebs7 | 1.00 | 0.00 | NA | 1.00 |
| dp1_sdq3 ~1 | 0.00 | 0.00 | NA | 0.00 |
| dp1_sdq8 ~1 | 0.00 | 0.00 | NA | 0.00 |
| dp1_sdq13 ~1 | 0.00 | 0.00 | NA | 0.00 |
| dp1_sdq16 ~1 | 0.00 | 0.00 | NA | 0.00 |
| dp1_sdq24 ~1 | 0.00 | 0.00 | NA | 0.00 |
| dp1_sdq5 ~1 | 0.00 | 0.00 | NA | 0.00 |
| dp1_sdq7_reversed ~1 | 0.00 | 0.00 | NA | 0.00 |
| dp1_sdq12 ~1 | 0.00 | 0.00 | NA | 0.00 |
| dp1_sdq18 ~1 | 0.00 | 0.00 | NA | 0.00 |
| dp1_sdq22 ~1 | 0.00 | 0.00 | NA | 0.00 |
| dp1_swemwebs1 ~1 | 0.00 | 0.00 | NA | 0.00 |
| dp1_swemwebs2 ~1 | 0.00 | 0.00 | NA | 0.00 |
| dp1_swemwebs3 ~1 | 0.00 | 0.00 | NA | 0.00 |
| dp1_swemwebs4 ~1 | 0.00 | 0.00 | NA | 0.00 |
| dp1_swemwebs5 ~1 | 0.00 | 0.00 | NA | 0.00 |
| dp1_swemwebs6 ~1 | 0.00 | 0.00 | NA | 0.00 |
| dp1_swemwebs7 ~1 | 0.00 | 0.00 | NA | 0.00 |
| Int ~1 | 0.00 | 0.00 | NA | 0.00 |
| Ext ~1 | 0.00 | 0.00 | NA | 0.00 |
| WB ~1 | 0.00 | 0.00 | NA | 0.00 |

**S3 Table C.** Factor loadings for bifactor g_wb_ with method factor model.

| Variable | Estimate | Standard Error | P value | Standardised Estimate |
| --- | --- | --- | --- | --- |
| Int =~ dp1_sdq3 | 1.00 | 0.00 | NA | 0.22 |
| Int =~ dp1_sdq8 | 2.88 | 0.17 | <0.001 | 0.62 |
| Int =~ dp1_sdq13 | 1.82 | 0.11 | <0.001 | 0.39 |
| Int =~ dp1_sdq16 | 2.18 | 0.13 | <0.001 | 0.47 |
| Int =~ dp1_sdq24 | 2.54 | 0.15 | <0.001 | 0.55 |
| Ext =~ dp1_sdq5 | 1.00 | 0.00 | NA | 0.47 |
| Ext =~ dp1_sdq7_reversed | 1.12 | 0.04 | <0.001 | 0.52 |
| Ext =~ dp1_sdq12 | 1.49 | 0.04 | <0.001 | 0.70 |
| Ext =~ dp1_sdq18 | 0.85 | 0.03 | <0.001 | 0.40 |
| Ext =~ dp1_sdq22 | 0.91 | 0.04 | <0.001 | 0.42 |
| WB =~ dp1_swemwebs1 | 1.00 | 0.00 | NA | 0.46 |
| WB =~ dp1_swemwebs2 | 0.93 | 0.06 | <0.001 | 0.43 |
| WB =~ dp1_swemwebs3 | 0.19 | 0.04 | <0.001 | 0.09 |
| WB =~ dp1_swemwebs4 | 0.26 | 0.04 | <0.001 | 0.12 |
| WB =~ dp1_swemwebs5 | 0.28 | 0.04 | <0.001 | 0.13 |
| WB =~ dp1_swemwebs6 | 0.72 | 0.05 | <0.001 | 0.33 |
| WB =~ dp1_swemwebs7 | 0.40 | 0.04 | <0.001 | 0.18 |
| c =~ dp1_sdq3 | 1.00 | 0.00 | NA | 0.33 |
| c =~ dp1_sdq8 | 1.05 | 0.03 | <0.001 | 0.34 |
| c =~ dp1_sdq13 | 1.67 | 0.05 | <0.001 | 0.54 |
| c =~ dp1_sdq16 | 0.90 | 0.03 | <0.001 | 0.29 |
| c =~ dp1_sdq24 | 0.77 | 0.03 | <0.001 | 0.25 |
| c =~ dp1_sdq5 | 1.27 | 0.04 | <0.001 | 0.41 |
| c =~ dp1_sdq7_reversed | 1.01 | 0.04 | <0.001 | 0.33 |
| c =~ dp1_sdq12 | 0.73 | 0.04 | <0.001 | 0.24 |
| c =~ dp1_sdq18 | 1.05 | 0.04 | <0.001 | 0.34 |
| c =~ dp1_sdq22 | 0.59 | 0.04 | <0.001 | 0.19 |
| c =~ dp1_swemwebs1 | -1.15 | 0.05 | <0.001 | -0.37 |
| c =~ dp1_swemwebs2 | -1.66 | 0.06 | <0.001 | -0.54 |
| c =~ dp1_swemwebs3 | -2.03 | 0.06 | <0.001 | -0.66 |
| c =~ dp1_swemwebs4 | -2.11 | 0.07 | <0.001 | -0.68 |
| c =~ dp1_swemwebs5 | -2.36 | 0.07 | <0.001 | -0.77 |
| c =~ dp1_swemwebs6 | -1.41 | 0.05 | <0.001 | -0.46 |
| c =~ dp1_swemwebs7 | -1.98 | 0.06 | <0.001 | -0.64 |
| Negativewording =~ dp1_sdq3 | 1.00 | 0.00 | NA | 0.33 |
| Negativewording =~ dp1_sdq5 | 1.00 | 0.00 | NA | 0.33 |
| Negativewording =~ dp1_sdq8 | 1.00 | 0.00 | NA | 0.33 |
| Negativewording =~ dp1_sdq12 | 1.00 | 0.00 | NA | 0.33 |
| Negativewording =~ dp1_sdq13 | 1.00 | 0.00 | NA | 0.33 |
| Negativewording =~ dp1_sdq16 | 1.00 | 0.00 | NA | 0.33 |
| Negativewording =~ dp1_sdq18 | 1.00 | 0.00 | NA | 0.33 |
| Negativewording =~ dp1_sdq22 | 1.00 | 0.00 | NA | 0.33 |
| Negativewording =~ dp1_sdq24 | 1.00 | 0.00 | NA | 0.33 |
| dp1_sdq3 \| t1 | -0.17 | 0.01 | <0.001 | -0.17 |
| dp1_sdq3 \| t2 | 0.95 | 0.01 | <0.001 | 0.95 |
| dp1_sdq8 \| t1 | -0.44 | 0.01 | <0.001 | -0.44 |
| dp1_sdq8 \| t2 | 0.62 | 0.01 | <0.001 | 0.62 |
| dp1_sdq13 \| t1 | 0.19 | 0.01 | <0.001 | 0.19 |
| dp1_sdq13 \| t2 | 1.24 | 0.01 | <0.001 | 1.24 |
| dp1_sdq16 \| t1 | -0.54 | 0.01 | <0.001 | -0.54 |
| dp1_sdq16 \| t2 | 0.58 | 0.01 | <0.001 | 0.58 |
| dp1_sdq24 \| t1 | 0.00 | 0.01 | 0.669 | 0.00 |
| dp1_sdq24 \| t2 | 1.05 | 0.01 | <0.001 | 1.05 |
| dp1_sdq5 \| t1 | -0.23 | 0.01 | <0.001 | -0.23 |
| dp1_sdq5 \| t2 | 0.77 | 0.01 | <0.001 | 0.77 |
| dp1_sdq7_reversed \| t1 | -0.14 | 0.01 | <0.001 | -0.14 |
| dp1_sdq7_reversed \| t2 | 1.54 | 0.02 | <0.001 | 1.54 |
| dp1_sdq12 \| t1 | 0.71 | 0.01 | <0.001 | 0.71 |
| dp1_sdq12 \| t2 | 1.66 | 0.02 | <0.001 | 1.66 |
| dp1_sdq18 \| t1 | 0.14 | 0.01 | <0.001 | 0.14 |
| dp1_sdq18 \| t2 | 1.05 | 0.01 | <0.001 | 1.05 |
| dp1_sdq22 \| t1 | 1.02 | 0.01 | <0.001 | 1.02 |
| dp1_sdq22 \| t2 | 1.77 | 0.02 | <0.001 | 1.77 |
| dp1_swemwebs1 \| t1 | -1.47 | 0.02 | <0.001 | -1.47 |
| dp1_swemwebs1 \| t2 | -0.78 | 0.01 | <0.001 | -0.78 |
| dp1_swemwebs1 \| t3 | 0.07 | 0.01 | <0.001 | 0.07 |
| dp1_swemwebs1 \| t4 | 0.99 | 0.01 | <0.001 | 0.99 |
| dp1_swemwebs2 \| t1 | -1.52 | 0.02 | <0.001 | -1.52 |
| dp1_swemwebs2 \| t2 | -0.74 | 0.01 | <0.001 | -0.74 |
| dp1_swemwebs2 \| t3 | 0.17 | 0.01 | <0.001 | 0.17 |
| dp1_swemwebs2 \| t4 | 1.17 | 0.01 | <0.001 | 1.17 |
| dp1_swemwebs3 \| t1 | -1.53 | 0.02 | <0.001 | -1.53 |
| dp1_swemwebs3 \| t2 | -0.68 | 0.01 | <0.001 | -0.68 |
| dp1_swemwebs3 \| t3 | 0.16 | 0.01 | <0.001 | 0.16 |
| dp1_swemwebs3 \| t4 | 1.02 | 0.01 | <0.001 | 1.02 |
| dp1_swemwebs4 \| t1 | -1.45 | 0.02 | <0.001 | -1.45 |
| dp1_swemwebs4 \| t2 | -0.75 | 0.01 | <0.001 | -0.75 |
| dp1_swemwebs4 \| t3 | 0.05 | 0.01 | <0.001 | 0.05 |
| dp1_swemwebs4 \| t4 | 0.92 | 0.01 | <0.001 | 0.92 |
| dp1_swemwebs5 \| t1 | -1.71 | 0.02 | <0.001 | -1.71 |
| dp1_swemwebs5 \| t2 | -0.96 | 0.01 | <0.001 | -0.96 |
| dp1_swemwebs5 \| t3 | -0.10 | 0.01 | <0.001 | -0.10 |
| dp1_swemwebs5 \| t4 | 0.83 | 0.01 | <0.001 | 0.83 |
| dp1_swemwebs6 \| t1 | -1.63 | 0.02 | <0.001 | -1.63 |
| dp1_swemwebs6 \| t2 | -0.97 | 0.01 | <0.001 | -0.97 |
| dp1_swemwebs6 \| t3 | -0.26 | 0.01 | <0.001 | -0.26 |
| dp1_swemwebs6 \| t4 | 0.57 | 0.01 | <0.001 | 0.57 |
| dp1_swemwebs7 \| t1 | -1.91 | 0.02 | <0.001 | -1.91 |
| dp1_swemwebs7 \| t2 | -1.27 | 0.01 | <0.001 | -1.27 |
| dp1_swemwebs7 \| t3 | -0.49 | 0.01 | <0.001 | -0.49 |
| dp1_swemwebs7 \| t4 | 0.40 | 0.01 | <0.001 | 0.40 |
| dp1_sdq3 ~~ dp1_sdq3 | 0.74 | 0.00 | NA | 0.74 |
| dp1_sdq8 ~~ dp1_sdq8 | 0.39 | 0.00 | NA | 0.39 |
| dp1_sdq13 ~~ dp1_sdq13 | 0.44 | 0.00 | NA | 0.44 |
| dp1_sdq16 ~~ dp1_sdq16 | 0.58 | 0.00 | NA | 0.58 |
| dp1_sdq24 ~~ dp1_sdq24 | 0.53 | 0.00 | NA | 0.53 |
| dp1_sdq5 ~~ dp1_sdq5 | 0.50 | 0.00 | NA | 0.50 |
| dp1_sdq7_reversed ~~ dp1_sdq7_reversed | 0.62 | 0.00 | NA | 0.62 |
| dp1_sdq12 ~~ dp1_sdq12 | 0.35 | 0.00 | NA | 0.35 |
| dp1_sdq18 ~~ dp1_sdq18 | 0.62 | 0.00 | NA | 0.62 |
| dp1_sdq22 ~~ dp1_sdq22 | 0.67 | 0.00 | NA | 0.67 |
| dp1_swemwebs1 ~~ dp1_swemwebs1 | 0.65 | 0.00 | NA | 0.65 |
| dp1_swemwebs2 ~~ dp1_swemwebs2 | 0.53 | 0.00 | NA | 0.53 |
| dp1_swemwebs3 ~~ dp1_swemwebs3 | 0.56 | 0.00 | NA | 0.56 |
| dp1_swemwebs4 ~~ dp1_swemwebs4 | 0.52 | 0.00 | NA | 0.52 |
| dp1_swemwebs5 ~~ dp1_swemwebs5 | 0.40 | 0.00 | NA | 0.40 |
| dp1_swemwebs6 ~~ dp1_swemwebs6 | 0.68 | 0.00 | NA | 0.68 |
| dp1_swemwebs7 ~~ dp1_swemwebs7 | 0.55 | 0.00 | NA | 0.55 |
| Int ~~ Int | 0.05 | 0.01 | <0.001 | 1.00 |
| Ext ~~ Ext | 0.22 | 0.01 | <0.001 | 1.00 |
| WB ~~ WB | 0.21 | 0.02 | <0.001 | 1.00 |
| c ~~ c | 0.11 | 0.01 | <0.001 | 1.00 |
| Negativewording ~~ Negativewording | 0.11 | 0.01 | <0.001 | 1.00 |
| Int ~~ Ext | 0.00 | 0.00 | NA | 0.00 |
| Int ~~ WB | 0.00 | 0.00 | NA | 0.00 |
| Int ~~ c | 0.00 | 0.00 | NA | 0.00 |
| Int ~~ Negativewording | 0.00 | 0.00 | NA | 0.00 |
| Ext ~~ WB | 0.00 | 0.00 | NA | 0.00 |
| Ext ~~ c | 0.00 | 0.00 | NA | 0.00 |
| Ext ~~ Negativewording | 0.00 | 0.00 | NA | 0.00 |
| WB ~~ c | 0.00 | 0.00 | NA | 0.00 |
| WB ~~ Negativewording | 0.00 | 0.00 | NA | 0.00 |
| c ~~ Negativewording | 0.00 | 0.00 | NA | 0.00 |
| dp1_sdq3 ~*~ dp1_sdq3 | 1.00 | 0.00 | NA | 1.00 |
| dp1_sdq8 ~*~ dp1_sdq8 | 1.00 | 0.00 | NA | 1.00 |
| dp1_sdq13 ~*~ dp1_sdq13 | 1.00 | 0.00 | NA | 1.00 |
| dp1_sdq16 ~*~ dp1_sdq16 | 1.00 | 0.00 | NA | 1.00 |
| dp1_sdq24 ~*~ dp1_sdq24 | 1.00 | 0.00 | NA | 1.00 |
| dp1_sdq5 ~*~ dp1_sdq5 | 1.00 | 0.00 | NA | 1.00 |
| dp1_sdq7_reversed ~*~ dp1_sdq7_reversed | 1.00 | 0.00 | NA | 1.00 |
| dp1_sdq12 ~*~ dp1_sdq12 | 1.00 | 0.00 | NA | 1.00 |
| dp1_sdq18 ~*~ dp1_sdq18 | 1.00 | 0.00 | NA | 1.00 |
| dp1_sdq22 ~*~ dp1_sdq22 | 1.00 | 0.00 | NA | 1.00 |
| dp1_swemwebs1 ~*~ dp1_swemwebs1 | 1.00 | 0.00 | NA | 1.00 |
| dp1_swemwebs2 ~*~ dp1_swemwebs2 | 1.00 | 0.00 | NA | 1.00 |
| dp1_swemwebs3 ~*~ dp1_swemwebs3 | 1.00 | 0.00 | NA | 1.00 |
| dp1_swemwebs4 ~*~ dp1_swemwebs4 | 1.00 | 0.00 | NA | 1.00 |
| dp1_swemwebs5 ~*~ dp1_swemwebs5 | 1.00 | 0.00 | NA | 1.00 |
| dp1_swemwebs6 ~*~ dp1_swemwebs6 | 1.00 | 0.00 | NA | 1.00 |
| dp1_swemwebs7 ~*~ dp1_swemwebs7 | 1.00 | 0.00 | NA | 1.00 |
| dp1_sdq3 ~1 | 0.00 | 0.00 | NA | 0.00 |
| dp1_sdq8 ~1 | 0.00 | 0.00 | NA | 0.00 |
| dp1_sdq13 ~1 | 0.00 | 0.00 | NA | 0.00 |
| dp1_sdq16 ~1 | 0.00 | 0.00 | NA | 0.00 |
| dp1_sdq24 ~1 | 0.00 | 0.00 | NA | 0.00 |
| dp1_sdq5 ~1 | 0.00 | 0.00 | NA | 0.00 |
| dp1_sdq7_reversed ~1 | 0.00 | 0.00 | NA | 0.00 |
| dp1_sdq12 ~1 | 0.00 | 0.00 | NA | 0.00 |
| dp1_sdq18 ~1 | 0.00 | 0.00 | NA | 0.00 |
| dp1_sdq22 ~1 | 0.00 | 0.00 | NA | 0.00 |
| dp1_swemwebs1 ~1 | 0.00 | 0.00 | NA | 0.00 |
| dp1_swemwebs2 ~1 | 0.00 | 0.00 | NA | 0.00 |
| dp1_swemwebs3 ~1 | 0.00 | 0.00 | NA | 0.00 |
| dp1_swemwebs4 ~1 | 0.00 | 0.00 | NA | 0.00 |
| dp1_swemwebs5 ~1 | 0.00 | 0.00 | NA | 0.00 |
| dp1_swemwebs6 ~1 | 0.00 | 0.00 | NA | 0.00 |
| dp1_swemwebs7 ~1 | 0.00 | 0.00 | NA | 0.00 |
| Int ~1 | 0.00 | 0.00 | NA | 0.00 |
| Ext ~1 | 0.00 | 0.00 | NA | 0.00 |
| WB ~1 | 0.00 | 0.00 | NA | 0.00 |
| c ~1 | 0.00 | 0.00 | NA | 0.00 |
| Negativewording ~1 | 0.00 | 0.00 | NA | 0.00 |

S3 Table D. Factor loadings for bifactor p model.

| Variable | Estimate | Standard Error | P value | Standardised Estimate |
| --- | --- | --- | --- | --- |
| Int =~ dp1_sdq3 | 1.00 | 0.00 | NA | 0.17 |
| Int =~ dp1_sdq8 | 3.67 | 0.32 | <0.001 | 0.64 |
| Int =~ dp1_sdq13 | 2.00 | 0.16 | <0.001 | 0.35 |
| Int =~ dp1_sdq16 | 3.41 | 0.31 | <0.001 | 0.59 |
| Int =~ dp1_sdq24 | 3.44 | 0.30 | <0.001 | 0.60 |
| Ext =~ dp1_sdq5 | 1.00 | 0.00 | NA | 0.49 |
| Ext =~ dp1_sdq7_reversed | 1.31 | 0.05 | <0.001 | 0.64 |
| Ext =~ dp1_sdq12 | 1.40 | 0.04 | <0.001 | 0.69 |
| Ext =~ dp1_sdq18 | 0.81 | 0.03 | <0.001 | 0.40 |
| Ext =~ dp1_sdq22 | 0.88 | 0.03 | <0.001 | 0.43 |
| p =~ dp1_sdq3 | 1.00 | 0.00 | NA | 0.54 |
| p =~ dp1_sdq8 | 0.79 | 0.03 | <0.001 | 0.43 |
| p =~ dp1_sdq13 | 1.33 | 0.04 | <0.001 | 0.72 |
| p =~ dp1_sdq16 | 0.57 | 0.03 | <0.001 | 0.31 |
| p =~ dp1_sdq24 | 0.64 | 0.03 | <0.001 | 0.35 |
| p =~ dp1_sdq5 | 1.01 | 0.04 | <0.001 | 0.55 |
| p =~ dp1_sdq7_reversed | 0.14 | 0.03 | <0.001 | 0.08 |
| p =~ dp1_sdq12 | 0.64 | 0.03 | <0.001 | 0.35 |
| p =~ dp1_sdq18 | 0.95 | 0.03 | <0.001 | 0.52 |
| p =~ dp1_sdq22 | 0.63 | 0.03 | <0.001 | 0.34 |
| dp1_sdq3 \| t1 | -0.17 | 0.01 | <0.001 | -0.17 |
| dp1_sdq3 \| t2 | 0.95 | 0.01 | <0.001 | 0.95 |
| dp1_sdq8 \| t1 | -0.44 | 0.01 | <0.001 | -0.44 |
| dp1_sdq8 \| t2 | 0.62 | 0.01 | <0.001 | 0.62 |
| dp1_sdq13 \| t1 | 0.19 | 0.01 | <0.001 | 0.19 |
| dp1_sdq13 \| t2 | 1.24 | 0.01 | <0.001 | 1.24 |
| dp1_sdq16 \| t1 | -0.54 | 0.01 | <0.001 | -0.54 |
| dp1_sdq16 \| t2 | 0.58 | 0.01 | <0.001 | 0.58 |
| dp1_sdq24 \| t1 | 0.00 | 0.01 | 0.668 | 0.00 |
| dp1_sdq24 \| t2 | 1.05 | 0.01 | <0.001 | 1.05 |
| dp1_sdq5 \| t1 | -0.23 | 0.01 | <0.001 | -0.23 |
| dp1_sdq5 \| t2 | 0.77 | 0.01 | <0.001 | 0.77 |
| dp1_sdq7_reversed \| t1 | -0.14 | 0.01 | <0.001 | -0.14 |
| dp1_sdq7_reversed \| t2 | 1.54 | 0.02 | <0.001 | 1.54 |
| dp1_sdq12 \| t1 | 0.71 | 0.01 | <0.001 | 0.71 |
| dp1_sdq12 \| t2 | 1.66 | 0.02 | <0.001 | 1.66 |
| dp1_sdq18 \| t1 | 0.14 | 0.01 | <0.001 | 0.14 |
| dp1_sdq18 \| t2 | 1.05 | 0.01 | <0.001 | 1.05 |
| dp1_sdq22 \| t1 | 1.02 | 0.01 | <0.001 | 1.02 |
| dp1_sdq22 \| t2 | 1.77 | 0.02 | <0.001 | 1.77 |
| dp1_sdq3 ~~ dp1_sdq3 | 0.67 | 0.00 | NA | 0.67 |
| dp1_sdq8 ~~ dp1_sdq8 | 0.41 | 0.00 | NA | 0.41 |
| dp1_sdq13 ~~ dp1_sdq13 | 0.35 | 0.00 | NA | 0.35 |
| dp1_sdq16 ~~ dp1_sdq16 | 0.55 | 0.00 | NA | 0.55 |
| dp1_sdq24 ~~ dp1_sdq24 | 0.52 | 0.00 | NA | 0.52 |
| dp1_sdq5 ~~ dp1_sdq5 | 0.46 | 0.00 | NA | 0.46 |
| dp1_sdq7_reversed ~~ dp1_sdq7_reversed | 0.58 | 0.00 | NA | 0.58 |
| dp1_sdq12 ~~ dp1_sdq12 | 0.40 | 0.00 | NA | 0.40 |
| dp1_sdq18 ~~ dp1_sdq18 | 0.58 | 0.00 | NA | 0.58 |
| dp1_sdq22 ~~ dp1_sdq22 | 0.70 | 0.00 | NA | 0.70 |
| Int ~~ Int | 0.03 | 0.01 | <0.001 | 1.00 |
| Ext ~~ Ext | 0.24 | 0.01 | <0.001 | 1.00 |
| p ~~ p | 0.30 | 0.01 | <0.001 | 1.00 |
| Int ~~ Ext | 0.00 | 0.00 | NA | 0.00 |
| Int ~~ p | 0.00 | 0.00 | NA | 0.00 |
| Ext ~~ p | 0.00 | 0.00 | NA | 0.00 |
| dp1_sdq3 ~*~ dp1_sdq3 | 1.00 | 0.00 | NA | 1.00 |
| dp1_sdq8 ~*~ dp1_sdq8 | 1.00 | 0.00 | NA | 1.00 |
| dp1_sdq13 ~*~ dp1_sdq13 | 1.00 | 0.00 | NA | 1.00 |
| dp1_sdq16 ~*~ dp1_sdq16 | 1.00 | 0.00 | NA | 1.00 |
| dp1_sdq24 ~*~ dp1_sdq24 | 1.00 | 0.00 | NA | 1.00 |
| dp1_sdq5 ~*~ dp1_sdq5 | 1.00 | 0.00 | NA | 1.00 |
| dp1_sdq7_reversed ~*~ dp1_sdq7_reversed | 1.00 | 0.00 | NA | 1.00 |
| dp1_sdq12 ~*~ dp1_sdq12 | 1.00 | 0.00 | NA | 1.00 |
| dp1_sdq18 ~*~ dp1_sdq18 | 1.00 | 0.00 | NA | 1.00 |
| dp1_sdq22 ~*~ dp1_sdq22 | 1.00 | 0.00 | NA | 1.00 |
| dp1_sdq3 ~1 | 0.00 | 0.00 | NA | 0.00 |
| dp1_sdq8 ~1 | 0.00 | 0.00 | NA | 0.00 |
| dp1_sdq13 ~1 | 0.00 | 0.00 | NA | 0.00 |
| dp1_sdq16 ~1 | 0.00 | 0.00 | NA | 0.00 |
| dp1_sdq24 ~1 | 0.00 | 0.00 | NA | 0.00 |
| dp1_sdq5 ~1 | 0.00 | 0.00 | NA | 0.00 |
| dp1_sdq7_reversed ~1 | 0.00 | 0.00 | NA | 0.00 |
| dp1_sdq12 ~1 | 0.00 | 0.00 | NA | 0.00 |
| dp1_sdq18 ~1 | 0.00 | 0.00 | NA | 0.00 |
| dp1_sdq22 ~1 | 0.00 | 0.00 | NA | 0.00 |
| Int ~1 | 0.00 | 0.00 | NA | 0.00 |
| Ext ~1 | 0.00 | 0.00 | NA | 0.00 |
| p ~1 | 0.00 | 0.00 | NA | 0.00 |

S3 Table E. Factor loadings for bifactor model with method factors.

| Variable | Estimate | Standard Error | P value | Standardised Estimate |
| --- | --- | --- | --- | --- |
| c =~ dp1_sdq3 | 1.00 | 0.00 | NA | 0.50 |
| c =~ dp1_sdq8 | 1.14 | 0.03 | <0.001 | 0.57 |
| c =~ dp1_sdq13 | 1.51 | 0.03 | <0.001 | 0.76 |
| c =~ dp1_sdq16 | 0.96 | 0.02 | <0.001 | 0.48 |
| c =~ dp1_sdq24 | 0.95 | 0.02 | <0.001 | 0.48 |
| c =~ dp1_sdq5 | 1.34 | 0.03 | <0.001 | 0.67 |
| c =~ dp1_sdq7_reversed | 0.62 | 0.02 | <0.001 | 0.31 |
| c =~ dp1_sdq12 | 1.12 | 0.03 | <0.001 | 0.56 |
| c =~ dp1_sdq18 | 1.16 | 0.03 | <0.001 | 0.58 |
| c =~ dp1_sdq22 | 0.87 | 0.03 | <0.001 | 0.44 |
| c =~ dp1_swemwebs1 | -0.41 | 0.02 | <0.001 | -0.21 |
| c =~ dp1_swemwebs2 | -0.61 | 0.02 | <0.001 | -0.31 |
| c =~ dp1_swemwebs3 | -0.85 | 0.02 | <0.001 | -0.43 |
| c =~ dp1_swemwebs4 | -0.85 | 0.02 | <0.001 | -0.43 |
| c =~ dp1_swemwebs5 | -0.93 | 0.02 | <0.001 | -0.47 |
| c =~ dp1_swemwebs6 | -0.49 | 0.02 | <0.001 | -0.25 |
| c =~ dp1_swemwebs7 | -0.77 | 0.02 | <0.001 | -0.39 |
| Positivewording =~ dp1_sdq7_reversed | 1.00 | 0.00 | NA | 0.22 |
| Positivewording =~ dp1_swemwebs1 | -2.12 | 0.11 | <0.001 | -0.47 |
| Positivewording =~ dp1_swemwebs2 | -2.62 | 0.13 | <0.001 | -0.58 |
| Positivewording =~ dp1_swemwebs3 | -2.13 | 0.10 | <0.001 | -0.47 |
| Positivewording =~ dp1_swemwebs4 | -2.40 | 0.12 | <0.001 | -0.53 |
| Positivewording =~ dp1_swemwebs5 | -2.71 | 0.13 | <0.001 | -0.60 |
| Positivewording =~ dp1_swemwebs6 | -2.27 | 0.11 | <0.001 | -0.50 |
| Positivewording =~ dp1_swemwebs7 | -2.49 | 0.12 | <0.001 | -0.55 |
| Negativewording =~ dp1_sdq3 | 1.00 | 0.00 | NA | 0.11 |
| Negativewording =~ dp1_sdq5 | -2.57 | 0.39 | <0.001 | -0.29 |
| Negativewording =~ dp1_sdq8 | 4.75 | 0.54 | <0.001 | 0.53 |
| Negativewording =~ dp1_sdq12 | -4.63 | 0.62 | <0.001 | -0.51 |
| Negativewording =~ dp1_sdq13 | 2.02 | 0.21 | <0.001 | 0.22 |
| Negativewording =~ dp1_sdq16 | 3.97 | 0.45 | <0.001 | 0.44 |
| Negativewording =~ dp1_sdq18 | -1.99 | 0.32 | <0.001 | -0.22 |
| Negativewording =~ dp1_sdq22 | -2.64 | 0.38 | <0.001 | -0.29 |
| Negativewording =~ dp1_sdq24 | 4.06 | 0.46 | <0.001 | 0.45 |
| dp1_sdq3 \| t1 | -0.17 | 0.01 | <0.001 | -0.17 |
| dp1_sdq3 \| t2 | 0.95 | 0.01 | <0.001 | 0.95 |
| dp1_sdq8 \| t1 | -0.44 | 0.01 | <0.001 | -0.44 |
| dp1_sdq8 \| t2 | 0.62 | 0.01 | <0.001 | 0.62 |
| dp1_sdq13 \| t1 | 0.19 | 0.01 | <0.001 | 0.19 |
| dp1_sdq13 \| t2 | 1.24 | 0.01 | <0.001 | 1.24 |
| dp1_sdq16 \| t1 | -0.54 | 0.01 | <0.001 | -0.54 |
| dp1_sdq16 \| t2 | 0.58 | 0.01 | <0.001 | 0.58 |
| dp1_sdq24 \| t1 | 0.00 | 0.01 | 0.669 | 0.00 |
| dp1_sdq24 \| t2 | 1.05 | 0.01 | <0.001 | 1.05 |
| dp1_sdq5 \| t1 | -0.23 | 0.01 | <0.001 | -0.23 |
| dp1_sdq5 \| t2 | 0.77 | 0.01 | <0.001 | 0.77 |
| dp1_sdq7_reversed \| t1 | -0.14 | 0.01 | <0.001 | -0.14 |
| dp1_sdq7_reversed \| t2 | 1.54 | 0.02 | <0.001 | 1.54 |
| dp1_sdq12 \| t1 | 0.71 | 0.01 | <0.001 | 0.71 |
| dp1_sdq12 \| t2 | 1.66 | 0.02 | <0.001 | 1.66 |
| dp1_sdq18 \| t1 | 0.14 | 0.01 | <0.001 | 0.14 |
| dp1_sdq18 \| t2 | 1.05 | 0.01 | <0.001 | 1.05 |
| dp1_sdq22 \| t1 | 1.02 | 0.01 | <0.001 | 1.02 |
| dp1_sdq22 \| t2 | 1.77 | 0.02 | <0.001 | 1.77 |
| dp1_swemwebs1 \| t1 | -1.47 | 0.02 | <0.001 | -1.47 |
| dp1_swemwebs1 \| t2 | -0.78 | 0.01 | <0.001 | -0.78 |
| dp1_swemwebs1 \| t3 | 0.07 | 0.01 | <0.001 | 0.07 |
| dp1_swemwebs1 \| t4 | 0.99 | 0.01 | <0.001 | 0.99 |
| dp1_swemwebs2 \| t1 | -1.52 | 0.02 | <0.001 | -1.52 |
| dp1_swemwebs2 \| t2 | -0.74 | 0.01 | <0.001 | -0.74 |
| dp1_swemwebs2 \| t3 | 0.17 | 0.01 | <0.001 | 0.17 |
| dp1_swemwebs2 \| t4 | 1.17 | 0.01 | <0.001 | 1.17 |
| dp1_swemwebs3 \| t1 | -1.53 | 0.02 | <0.001 | -1.53 |
| dp1_swemwebs3 \| t2 | -0.68 | 0.01 | <0.001 | -0.68 |
| dp1_swemwebs3 \| t3 | 0.16 | 0.01 | <0.001 | 0.16 |
| dp1_swemwebs3 \| t4 | 1.02 | 0.01 | <0.001 | 1.02 |
| dp1_swemwebs4 \| t1 | -1.45 | 0.02 | <0.001 | -1.45 |
| dp1_swemwebs4 \| t2 | -0.75 | 0.01 | <0.001 | -0.75 |
| dp1_swemwebs4 \| t3 | 0.05 | 0.01 | <0.001 | 0.05 |
| dp1_swemwebs4 \| t4 | 0.92 | 0.01 | <0.001 | 0.92 |
| dp1_swemwebs5 \| t1 | -1.71 | 0.02 | <0.001 | -1.71 |
| dp1_swemwebs5 \| t2 | -0.96 | 0.01 | <0.001 | -0.96 |
| dp1_swemwebs5 \| t3 | -0.10 | 0.01 | <0.001 | -0.10 |
| dp1_swemwebs5 \| t4 | 0.83 | 0.01 | <0.001 | 0.83 |
| dp1_swemwebs6 \| t1 | -1.63 | 0.02 | <0.001 | -1.63 |
| dp1_swemwebs6 \| t2 | -0.97 | 0.01 | <0.001 | -0.97 |
| dp1_swemwebs6 \| t3 | -0.26 | 0.01 | <0.001 | -0.26 |
| dp1_swemwebs6 \| t4 | 0.57 | 0.01 | <0.001 | 0.57 |
| dp1_swemwebs7 \| t1 | -1.91 | 0.02 | <0.001 | -1.91 |
| dp1_swemwebs7 \| t2 | -1.27 | 0.01 | <0.001 | -1.27 |
| dp1_swemwebs7 \| t3 | -0.49 | 0.01 | <0.001 | -0.49 |
| dp1_swemwebs7 \| t4 | 0.40 | 0.01 | <0.001 | 0.40 |
| dp1_sdq3 ~~ dp1_sdq3 | 0.73 | 0.00 | NA | 0.73 |
| dp1_sdq8 ~~ dp1_sdq8 | 0.39 | 0.00 | NA | 0.39 |
| dp1_sdq13 ~~ dp1_sdq13 | 0.37 | 0.00 | NA | 0.37 |
| dp1_sdq16 ~~ dp1_sdq16 | 0.57 | 0.00 | NA | 0.57 |
| dp1_sdq24 ~~ dp1_sdq24 | 0.57 | 0.00 | NA | 0.57 |
| dp1_sdq5 ~~ dp1_sdq5 | 0.46 | 0.00 | NA | 0.46 |
| dp1_sdq7_reversed ~~ dp1_sdq7_reversed | 0.85 | 0.00 | NA | 0.85 |
| dp1_sdq12 ~~ dp1_sdq12 | 0.42 | 0.00 | NA | 0.42 |
| dp1_sdq18 ~~ dp1_sdq18 | 0.61 | 0.00 | NA | 0.61 |
| dp1_sdq22 ~~ dp1_sdq22 | 0.72 | 0.00 | NA | 0.72 |
| dp1_swemwebs1 ~~ dp1_swemwebs1 | 0.74 | 0.00 | NA | 0.74 |
| dp1_swemwebs2 ~~ dp1_swemwebs2 | 0.57 | 0.00 | NA | 0.57 |
| dp1_swemwebs3 ~~ dp1_swemwebs3 | 0.59 | 0.00 | NA | 0.59 |
| dp1_swemwebs4 ~~ dp1_swemwebs4 | 0.54 | 0.00 | NA | 0.54 |
| dp1_swemwebs5 ~~ dp1_swemwebs5 | 0.42 | 0.00 | NA | 0.42 |
| dp1_swemwebs6 ~~ dp1_swemwebs6 | 0.69 | 0.00 | NA | 0.69 |
| dp1_swemwebs7 ~~ dp1_swemwebs7 | 0.55 | 0.00 | NA | 0.55 |
| c ~~ c | 0.25 | 0.01 | <0.001 | 1.00 |
| Positivewording ~~ Positivewording | 0.05 | 0.00 | <0.001 | 1.00 |
| Negativewording ~~ Negativewording | 0.01 | 0.00 | <0.001 | 1.00 |
| c ~~ Positivewording | 0.00 | 0.00 | NA | 0.00 |
| c ~~ Negativewording | 0.00 | 0.00 | NA | 0.00 |
| Positivewording ~~ Negativewording | 0.00 | 0.00 | NA | 0.00 |
| dp1_sdq3 ~*~ dp1_sdq3 | 1.00 | 0.00 | NA | 1.00 |
| dp1_sdq8 ~*~ dp1_sdq8 | 1.00 | 0.00 | NA | 1.00 |
| dp1_sdq13 ~*~ dp1_sdq13 | 1.00 | 0.00 | NA | 1.00 |
| dp1_sdq16 ~*~ dp1_sdq16 | 1.00 | 0.00 | NA | 1.00 |
| dp1_sdq24 ~*~ dp1_sdq24 | 1.00 | 0.00 | NA | 1.00 |
| dp1_sdq5 ~*~ dp1_sdq5 | 1.00 | 0.00 | NA | 1.00 |
| dp1_sdq7_reversed ~*~ dp1_sdq7_reversed | 1.00 | 0.00 | NA | 1.00 |
| dp1_sdq12 ~*~ dp1_sdq12 | 1.00 | 0.00 | NA | 1.00 |
| dp1_sdq18 ~*~ dp1_sdq18 | 1.00 | 0.00 | NA | 1.00 |
| dp1_sdq22 ~*~ dp1_sdq22 | 1.00 | 0.00 | NA | 1.00 |
| dp1_swemwebs1 ~*~ dp1_swemwebs1 | 1.00 | 0.00 | NA | 1.00 |
| dp1_swemwebs2 ~*~ dp1_swemwebs2 | 1.00 | 0.00 | NA | 1.00 |
| dp1_swemwebs3 ~*~ dp1_swemwebs3 | 1.00 | 0.00 | NA | 1.00 |
| dp1_swemwebs4 ~*~ dp1_swemwebs4 | 1.00 | 0.00 | NA | 1.00 |
| dp1_swemwebs5 ~*~ dp1_swemwebs5 | 1.00 | 0.00 | NA | 1.00 |
| dp1_swemwebs6 ~*~ dp1_swemwebs6 | 1.00 | 0.00 | NA | 1.00 |
| dp1_swemwebs7 ~*~ dp1_swemwebs7 | 1.00 | 0.00 | NA | 1.00 |
| dp1_sdq3 ~1 | 0.00 | 0.00 | NA | 0.00 |
| dp1_sdq8 ~1 | 0.00 | 0.00 | NA | 0.00 |
| dp1_sdq13 ~1 | 0.00 | 0.00 | NA | 0.00 |
| dp1_sdq16 ~1 | 0.00 | 0.00 | NA | 0.00 |
| dp1_sdq24 ~1 | 0.00 | 0.00 | NA | 0.00 |
| dp1_sdq5 ~1 | 0.00 | 0.00 | NA | 0.00 |
| dp1_sdq7_reversed ~1 | 0.00 | 0.00 | NA | 0.00 |
| dp1_sdq12 ~1 | 0.00 | 0.00 | NA | 0.00 |
| dp1_sdq18 ~1 | 0.00 | 0.00 | NA | 0.00 |
| dp1_sdq22 ~1 | 0.00 | 0.00 | NA | 0.00 |
| dp1_swemwebs1 ~1 | 0.00 | 0.00 | NA | 0.00 |
| dp1_swemwebs2 ~1 | 0.00 | 0.00 | NA | 0.00 |
| dp1_swemwebs3 ~1 | 0.00 | 0.00 | NA | 0.00 |
| dp1_swemwebs4 ~1 | 0.00 | 0.00 | NA | 0.00 |
| dp1_swemwebs5 ~1 | 0.00 | 0.00 | NA | 0.00 |
| dp1_swemwebs6 ~1 | 0.00 | 0.00 | NA | 0.00 |
| dp1_swemwebs7 ~1 | 0.00 | 0.00 | NA | 0.00 |
| c ~1 | 0.00 | 0.00 | NA | 0.00 |
| Positivewording ~1 | 0.00 | 0.00 | NA | 0.00 |
| Negativewording ~1 | 0.00 | 0.00 | NA | 0.00 |
