# Supplementary material for: Incorporating wellbeing into general factor models: A more complete mental state?
Source: PLoS One. 2025 Nov 17;20(11):e0335657. doi: 10.1371/journal.pone.0335657 (PMC12622774; doi:10.1371/journal.pone.0335657)
Supplement: S4 Table — (DOCX) [file pone.0335657.s004.docx]

**S4 Table. Factor loadings and parameter estimates for structural models**

**Table S4 A.** Unstandardized parameter estimates for bifactor g_wb_ with method factor mediation model.

| Variable | Estimate | Standard Error | P value |
| --- | --- | --- | --- |
| Int =~ dp1_sdq3 | 1.00 | 0.00 | NA |
| Int =~ dp1_sdq8 | 3.11 | 0.22 | <0.001 |
| Int =~ dp1_sdq13 | 1.90 | 0.13 | <0.001 |
| Int =~ dp1_sdq16 | 2.37 | 0.17 | <0.001 |
| Int =~ dp1_sdq24 | 2.74 | 0.19 | <0.001 |
| Ext =~ dp1_sdq5 | 1.00 | 0.00 | NA |
| Ext =~ dp1_sdq7_reversed | 1.08 | 0.04 | <0.001 |
| Ext =~ dp1_sdq12 | 1.66 | 0.06 | <0.001 |
| Ext =~ dp1_sdq18 | 0.79 | 0.03 | <0.001 |
| Ext =~ dp1_sdq22 | 0.90 | 0.04 | <0.001 |
| WB =~ dp1_swemwebs1 | 1.00 | 0.00 | NA |
| WB =~ dp1_swemwebs2 | 1.03 | 0.04 | <0.001 |
| WB =~ dp1_swemwebs3 | 0.48 | 0.03 | <0.001 |
| WB =~ dp1_swemwebs4 | 0.61 | 0.03 | <0.001 |
| WB =~ dp1_swemwebs5 | 0.69 | 0.03 | <0.001 |
| WB =~ dp1_swemwebs6 | 0.88 | 0.04 | <0.001 |
| WB =~ dp1_swemwebs7 | 0.75 | 0.03 | <0.001 |
| c =~ dp1_sdq3 | 1.00 | 0.00 | NA |
| c =~ dp1_sdq8 | 1.08 | 0.04 | <0.001 |
| c =~ dp1_sdq13 | 1.66 | 0.05 | <0.001 |
| c =~ dp1_sdq16 | 0.86 | 0.03 | <0.001 |
| c =~ dp1_sdq24 | 0.75 | 0.03 | <0.001 |
| c =~ dp1_sdq5 | 1.30 | 0.04 | <0.001 |
| c =~ dp1_sdq7_reversed | 0.93 | 0.04 | <0.001 |
| c =~ dp1_sdq12 | 0.72 | 0.04 | <0.001 |
| c =~ dp1_sdq18 | 1.13 | 0.04 | <0.001 |
| c =~ dp1_sdq22 | 0.60 | 0.04 | <0.001 |
| c =~ dp1_swemwebs1 | -0.87 | 0.05 | <0.001 |
| c =~ dp1_swemwebs2 | -1.35 | 0.05 | <0.001 |
| c =~ dp1_swemwebs3 | -1.77 | 0.06 | <0.001 |
| c =~ dp1_swemwebs4 | -1.77 | 0.06 | <0.001 |
| c =~ dp1_swemwebs5 | -1.95 | 0.07 | <0.001 |
| c =~ dp1_swemwebs6 | -1.14 | 0.05 | <0.001 |
| c =~ dp1_swemwebs7 | -1.62 | 0.06 | <0.001 |
| Neg_word =~ dp1_sdq3 | 1.00 | 0.00 | NA |
| Neg_word =~ dp1_sdq5 | 1.00 | 0.00 | NA |
| Neg_word =~ dp1_sdq8 | 1.00 | 0.00 | NA |
| Neg_word =~ dp1_sdq12 | 1.00 | 0.00 | NA |
| Neg_word =~ dp1_sdq13 | 1.00 | 0.00 | NA |
| Neg_word =~ dp1_sdq16 | 1.00 | 0.00 | NA |
| Neg_word =~ dp1_sdq18 | 1.00 | 0.00 | NA |
| Neg_word =~ dp1_sdq22 | 1.00 | 0.00 | NA |
| Neg_word =~ dp1_sdq24 | 1.00 | 0.00 | NA |
| rdp3_impactscore ~ c | 2.51 | 0.16 | <0.001 |
| rdp3_impactscore ~ dp1_impactscore | 0.13 | 0.02 | <0.001 |
| rdp3_impactscore ~ gender1 | -0.93 | 0.05 | <0.001 |
| rdp3_impactscore ~ npd_everfsm1 | 0.47 | 0.05 | <0.001 |
| rdp3_impactscore ~ npd_all_sen1 | 0.38 | 0.07 | <0.001 |
| rdp3_impactscore ~ asian | -0.78 | 0.09 | <0.001 |
| rdp3_impactscore ~ black | -0.81 | 0.11 | <0.001 |
| rdp3_impactscore ~ mixed | 0.02 | 0.11 | 0.87 |
| rdp3_impactscore ~ other | -0.37 | 0.14 | <0.01 |
| rdp3_impactscore ~ IDACIScore | 0.19 | 0.17 | 0.25 |
| rdp3_impactscore ~ Int | 1.66 | 0.22 | <0.001 |
| rdp3_impactscore ~ Ext | 0.39 | 0.08 | <0.001 |
| rdp3_impactscore ~ WB | 0.36 | 0.11 | <0.01 |
| dp1_impactscore ~ c | 4.49 | 0.15 | <0.001 |
| dp1_impactscore ~ gender1 | -0.18 | 0.05 | <0.001 |
| dp1_impactscore ~ npd_everfsm1 | 0.46 | 0.05 | <0.001 |
| dp1_impactscore ~ npd_all_sen1 | 0.80 | 0.06 | <0.001 |
| dp1_impactscore ~ asian | -0.54 | 0.09 | <0.001 |
| dp1_impactscore ~ black | -0.36 | 0.10 | <0.001 |
| dp1_impactscore ~ mixed | -0.10 | 0.11 | 0.376 |
| dp1_impactscore ~ other | -0.16 | 0.12 | 0.182 |
| dp1_impactscore ~ IDACIScore | 0.38 | 0.16 | <0.05 |
| dp1_impactscore ~ Int | 3.67 | 0.31 | <0.001 |
| dp1_impactscore ~ Ext | 0.90 | 0.08 | <0.001 |
| dp1_impactscore ~ WB | 0.80 | 0.11 | <0.001 |
| dp1_sdq3 \| t1 | -0.15 | 0.02 | <0.001 |
| dp1_sdq3 \| t2 | 0.99 | 0.02 | <0.001 |
| dp1_sdq8 \| t1 | -0.70 | 0.02 | <0.001 |
| dp1_sdq8 \| t2 | 0.38 | 0.02 | <0.001 |
| dp1_sdq13 \| t1 | 0.19 | 0.02 | <0.001 |
| dp1_sdq13 \| t2 | 1.26 | 0.02 | <0.001 |
| dp1_sdq16 \| t1 | -0.66 | 0.02 | <0.001 |
| dp1_sdq16 \| t2 | 0.48 | 0.02 | <0.001 |
| dp1_sdq24 \| t1 | -0.13 | 0.02 | <0.001 |
| dp1_sdq24 \| t2 | 0.93 | 0.02 | <0.001 |
| dp1_sdq5 \| t1 | 0.10 | 0.02 | <0.001 |
| dp1_sdq5 \| t2 | 1.13 | 0.02 | <0.001 |
| dp1_sdq7_reversed \| t1 | 0.10 | 0.02 | <0.001 |
| dp1_sdq7_reversed \| t2 | 1.80 | 0.03 | <0.001 |
| dp1_sdq12 \| t1 | 1.04 | 0.03 | <0.001 |
| dp1_sdq12 \| t2 | 2.01 | 0.03 | <0.001 |
| dp1_sdq18 \| t1 | 0.47 | 0.02 | <0.001 |
| dp1_sdq18 \| t2 | 1.41 | 0.02 | <0.001 |
| dp1_sdq22 \| t1 | 1.32 | 0.03 | <0.001 |
| dp1_sdq22 \| t2 | 2.09 | 0.03 | <0.001 |
| dp1_swemwebs1 \| t1 | -1.53 | 0.03 | <0.001 |
| dp1_swemwebs1 \| t2 | -0.83 | 0.02 | <0.001 |
| dp1_swemwebs1 \| t3 | 0.02 | 0.02 | 0.317 |
| dp1_swemwebs1 \| t4 | 0.95 | 0.02 | <0.001 |
| dp1_swemwebs2 \| t1 | -1.52 | 0.03 | <0.001 |
| dp1_swemwebs2 \| t2 | -0.74 | 0.02 | <0.001 |
| dp1_swemwebs2 \| t3 | 0.18 | 0.02 | <0.001 |
| dp1_swemwebs2 \| t4 | 1.18 | 0.02 | <0.001 |
| dp1_swemwebs3 \| t1 | -1.43 | 0.03 | <0.001 |
| dp1_swemwebs3 \| t2 | -0.57 | 0.02 | <0.001 |
| dp1_swemwebs3 \| t3 | 0.27 | 0.02 | <0.001 |
| dp1_swemwebs3 \| t4 | 1.14 | 0.02 | <0.001 |
| dp1_swemwebs4 \| t1 | -1.55 | 0.03 | <0.001 |
| dp1_swemwebs4 \| t2 | -0.84 | 0.02 | <0.001 |
| dp1_swemwebs4 \| t3 | -0.04 | 0.02 | 0.056 |
| dp1_swemwebs4 \| t4 | 0.84 | 0.02 | <0.001 |
| dp1_swemwebs5 \| t1 | -1.73 | 0.03 | <0.001 |
| dp1_swemwebs5 \| t2 | -0.97 | 0.02 | <0.001 |
| dp1_swemwebs5 \| t3 | -0.11 | 0.02 | <0.001 |
| dp1_swemwebs5 \| t4 | 0.82 | 0.02 | <0.001 |
| dp1_swemwebs6 \| t1 | -1.80 | 0.03 | <0.001 |
| dp1_swemwebs6 \| t2 | -1.13 | 0.02 | <0.001 |
| dp1_swemwebs6 \| t3 | -0.42 | 0.02 | <0.001 |
| dp1_swemwebs6 \| t4 | 0.42 | 0.02 | <0.001 |
| dp1_swemwebs7 \| t1 | -1.90 | 0.03 | <0.001 |
| dp1_swemwebs7 \| t2 | -1.26 | 0.02 | <0.001 |
| dp1_swemwebs7 \| t3 | -0.48 | 0.02 | <0.001 |
| dp1_swemwebs7 \| t4 | 0.42 | 0.02 | <0.001 |
| dp1_sdq3 ~~ dp1_sdq3 | 0.74 | 0.00 | NA |
| dp1_sdq8 ~~ dp1_sdq8 | 0.40 | 0.00 | NA |
| dp1_sdq13 ~~ dp1_sdq13 | 0.43 | 0.00 | NA |
| dp1_sdq16 ~~ dp1_sdq16 | 0.60 | 0.00 | NA |
| dp1_sdq24 ~~ dp1_sdq24 | 0.56 | 0.00 | NA |
| dp1_sdq5 ~~ dp1_sdq5 | 0.51 | 0.00 | NA |
| dp1_sdq7_reversed ~~ dp1_sdq7_reversed | 0.68 | 0.00 | NA |
| dp1_sdq12 ~~ dp1_sdq12 | 0.33 | 0.00 | NA |
| dp1_sdq18 ~~ dp1_sdq18 | 0.63 | 0.00 | NA |
| dp1_sdq22 ~~ dp1_sdq22 | 0.71 | 0.00 | NA |
| dp1_swemwebs1 ~~ dp1_swemwebs1 | 0.70 | 0.00 | NA |
| dp1_swemwebs2 ~~ dp1_swemwebs2 | 0.55 | 0.00 | NA |
| dp1_swemwebs3 ~~ dp1_swemwebs3 | 0.56 | 0.00 | NA |
| dp1_swemwebs4 ~~ dp1_swemwebs4 | 0.53 | 0.00 | NA |
| dp1_swemwebs5 ~~ dp1_swemwebs5 | 0.43 | 0.00 | NA |
| dp1_swemwebs6 ~~ dp1_swemwebs6 | 0.68 | 0.00 | NA |
| dp1_swemwebs7 ~~ dp1_swemwebs7 | 0.56 | 0.00 | NA |
| rdp3_impactscore ~~ rdp3_impactscore | 6.12 | 0.09 | <0.001 |
| dp1_impactscore ~~ dp1_impactscore | 3.68 | 0.08 | <0.001 |
| Int ~~ Int | 0.04 | 0.01 | <0.001 |
| Ext ~~ Ext | 0.18 | 0.01 | <0.001 |
| WB ~~ WB | 0.21 | 0.01 | <0.001 |
| c ~~ c | 0.12 | 0.01 | <0.001 |
| Neg_word ~~ Neg_word | 0.10 | 0.01 | <0.001 |
| Int ~~ Ext | 0.00 | 0.00 | NA |
| Int ~~ WB | 0.00 | 0.00 | NA |
| Int ~~ c | 0.00 | 0.00 | NA |
| Int ~~ Neg_word | 0.00 | 0.00 | NA |
| Ext ~~ WB | 0.00 | 0.00 | NA |
| Ext ~~ c | 0.00 | 0.00 | NA |
| Ext ~~ Neg_word | 0.00 | 0.00 | NA |
| WB ~~ c | 0.00 | 0.00 | NA |
| WB ~~ Neg_word | 0.00 | 0.00 | NA |
| c ~~ Neg_word | 0.00 | 0.00 | NA |
| gender1 ~~ gender1 | 0.25 | 0.00 | NA |
| gender1 ~~ npd_everfsm1 | 0.00 | 0.00 | NA |
| gender1 ~~ npd_all_sen1 | 0.02 | 0.00 | NA |
| gender1 ~~ asian | -0.01 | 0.00 | NA |
| gender1 ~~ black | 0.00 | 0.00 | NA |
| gender1 ~~ mixed | 0.00 | 0.00 | NA |
| gender1 ~~ other | 0.00 | 0.00 | NA |
| gender1 ~~ IDACIScore | 0.00 | 0.00 | NA |
| npd_everfsm1 ~~ npd_everfsm1 | 0.23 | 0.00 | NA |
| npd_everfsm1 ~~ npd_all_sen1 | 0.02 | 0.00 | NA |
| npd_everfsm1 ~~ asian | 0.00 | 0.00 | NA |
| npd_everfsm1 ~~ black | 0.01 | 0.00 | NA |
| npd_everfsm1 ~~ mixed | 0.01 | 0.00 | NA |
| npd_everfsm1 ~~ other | 0.00 | 0.00 | NA |
| npd_everfsm1 ~~ IDACIScore | 0.02 | 0.00 | NA |
| npd_all_sen1 ~~ npd_all_sen1 | 0.11 | 0.00 | NA |
| npd_all_sen1 ~~ asian | 0.00 | 0.00 | NA |
| npd_all_sen1 ~~ black | 0.00 | 0.00 | NA |
| npd_all_sen1 ~~ mixed | 0.00 | 0.00 | NA |
| npd_all_sen1 ~~ other | 0.00 | 0.00 | NA |
| npd_all_sen1 ~~ IDACIScore | 0.00 | 0.00 | NA |
| asian ~~ asian | 0.09 | 0.00 | NA |
| asian ~~ black | -0.01 | 0.00 | NA |
| asian ~~ mixed | 0.00 | 0.00 | NA |
| asian ~~ other | 0.00 | 0.00 | NA |
| asian ~~ IDACIScore | 0.00 | 0.00 | NA |
| black ~~ black | 0.06 | 0.00 | NA |
| black ~~ mixed | 0.00 | 0.00 | NA |
| black ~~ other | 0.00 | 0.00 | NA |
| black ~~ IDACIScore | 0.00 | 0.00 | NA |
| mixed ~~ mixed | 0.04 | 0.00 | NA |
| mixed ~~ other | 0.00 | 0.00 | NA |
| mixed ~~ IDACIScore | 0.00 | 0.00 | NA |
| other ~~ other | 0.03 | 0.00 | NA |
| other ~~ IDACIScore | 0.00 | 0.00 | NA |
| IDACIScore ~~ IDACIScore | 0.02 | 0.00 | NA |
| dp1_sdq3 ~*~ dp1_sdq3 | 1.00 | 0.00 | NA |
| dp1_sdq8 ~*~ dp1_sdq8 | 1.00 | 0.00 | NA |
| dp1_sdq13 ~*~ dp1_sdq13 | 1.00 | 0.00 | NA |
| dp1_sdq16 ~*~ dp1_sdq16 | 1.00 | 0.00 | NA |
| dp1_sdq24 ~*~ dp1_sdq24 | 1.00 | 0.00 | NA |
| dp1_sdq5 ~*~ dp1_sdq5 | 1.00 | 0.00 | NA |
| dp1_sdq7_reversed ~*~ dp1_sdq7_reversed | 1.00 | 0.00 | NA |
| dp1_sdq12 ~*~ dp1_sdq12 | 1.00 | 0.00 | NA |
| dp1_sdq18 ~*~ dp1_sdq18 | 1.00 | 0.00 | NA |
| dp1_sdq22 ~*~ dp1_sdq22 | 1.00 | 0.00 | NA |
| dp1_swemwebs1 ~*~ dp1_swemwebs1 | 1.00 | 0.00 | NA |
| dp1_swemwebs2 ~*~ dp1_swemwebs2 | 1.00 | 0.00 | NA |
| dp1_swemwebs3 ~*~ dp1_swemwebs3 | 1.00 | 0.00 | NA |
| dp1_swemwebs4 ~*~ dp1_swemwebs4 | 1.00 | 0.00 | NA |
| dp1_swemwebs5 ~*~ dp1_swemwebs5 | 1.00 | 0.00 | NA |
| dp1_swemwebs6 ~*~ dp1_swemwebs6 | 1.00 | 0.00 | NA |
| dp1_swemwebs7 ~*~ dp1_swemwebs7 | 1.00 | 0.00 | NA |
| dp1_sdq3 ~1 | 0.00 | 0.00 | NA |
| dp1_sdq8 ~1 | 0.00 | 0.00 | NA |
| dp1_sdq13 ~1 | 0.00 | 0.00 | NA |
| dp1_sdq16 ~1 | 0.00 | 0.00 | NA |
| dp1_sdq24 ~1 | 0.00 | 0.00 | NA |
| dp1_sdq5 ~1 | 0.00 | 0.00 | NA |
| dp1_sdq7_reversed ~1 | 0.00 | 0.00 | NA |
| dp1_sdq12 ~1 | 0.00 | 0.00 | NA |
| dp1_sdq18 ~1 | 0.00 | 0.00 | NA |
| dp1_sdq22 ~1 | 0.00 | 0.00 | NA |
| dp1_swemwebs1 ~1 | 0.00 | 0.00 | NA |
| dp1_swemwebs2 ~1 | 0.00 | 0.00 | NA |
| dp1_swemwebs3 ~1 | 0.00 | 0.00 | NA |
| dp1_swemwebs4 ~1 | 0.00 | 0.00 | NA |
| dp1_swemwebs5 ~1 | 0.00 | 0.00 | NA |
| dp1_swemwebs6 ~1 | 0.00 | 0.00 | NA |
| dp1_swemwebs7 ~1 | 0.00 | 0.00 | NA |
| rdp3_impactscore ~1 | 2.49 | 0.06 | <0.001 |
| dp1_impactscore ~1 | 1.89 | 0.06 | <0.001 |
| gender1 ~1 | 0.47 | 0.00 | NA |
| npd_everfsm1 ~1 | 0.37 | 0.00 | NA |
| npd_all_sen1 ~1 | 0.13 | 0.00 | NA |
| asian ~1 | 0.10 | 0.00 | NA |
| black ~1 | 0.06 | 0.00 | NA |
| mixed ~1 | 0.04 | 0.00 | NA |
| other ~1 | 0.03 | 0.00 | NA |
| IDACIScore ~1 | 0.25 | 0.00 | NA |
| Int ~1 | 0.00 | 0.00 | NA |
| Ext ~1 | 0.00 | 0.00 | NA |
| WB ~1 | 0.00 | 0.00 | NA |
| c ~1 | 0.00 | 0.00 | NA |
| Neg_word ~1 | 0.00 | 0.00 | NA |
| direct := a | 2.51 | 0.16 | <0.001 |
| indirect := d*b | 0.59 | 0.07 | <0.001 |
| total := (d*b)+a | 3.10 | 0.13 | <0.001 |

**Table S4 B.** Unstandardized parameter estimates for correlated factors mediation model.

| Variable | Estimate | Standard Error | P value |
| --- | --- | --- | --- |
| Int =~ dp1_sdq3 | 1.00 | 0.00 | NA |
| Int =~ dp1_sdq8 | 1.33 | 0.03 | <0.001 |
| Int =~ dp1_sdq13 | 1.61 | 0.03 | <0.001 |
| Int =~ dp1_sdq16 | 1.10 | 0.02 | <0.001 |
| Int =~ dp1_sdq24 | 1.09 | 0.03 | <0.001 |
| Ext =~ dp1_sdq5 | 1.00 | 0.00 | NA |
| Ext =~ dp1_sdq7_reversed | 0.65 | 0.02 | <0.001 |
| Ext =~ dp1_sdq12 | 0.80 | 0.02 | <0.001 |
| Ext =~ dp1_sdq18 | 0.87 | 0.02 | <0.001 |
| Ext =~ dp1_sdq22 | 0.62 | 0.02 | <0.001 |
| WB =~ dp1_swemwebs1 | 1.00 | 0.00 | NA |
| WB =~ dp1_swemwebs2 | 1.36 | 0.02 | <0.001 |
| WB =~ dp1_swemwebs3 | 1.47 | 0.03 | <0.001 |
| WB =~ dp1_swemwebs4 | 1.52 | 0.03 | <0.001 |
| WB =~ dp1_swemwebs5 | 1.69 | 0.03 | <0.001 |
| WB =~ dp1_swemwebs6 | 1.16 | 0.02 | <0.001 |
| WB =~ dp1_swemwebs7 | 1.47 | 0.03 | <0.001 |
| rdp3_impactscore ~ dp1_impactscore | 0.23 | 0.01 | <0.001 |
| rdp3_impactscore ~ gender | -0.91 | 0.05 | <0.001 |
| rdp3_impactscore ~ asian | -0.72 | 0.09 | <0.001 |
| rdp3_impactscore ~ black | -0.78 | 0.11 | <0.001 |
| rdp3_impactscore ~ mixed | 0.03 | 0.11 | 0.8 |
| rdp3_impactscore ~ other | -0.35 | 0.14 | <0.01 |
| rdp3_impactscore ~ npd_everfsm | 0.43 | 0.05 | <0.001 |
| rdp3_impactscore ~ npd_all_sen | 0.30 | 0.07 | <0.001 |
| rdp3_impactscore ~ IDACIScore | 0.16 | 0.16 | 0.342 |
| rdp3_impactscore ~ Int | 0.76 | 0.07 | <0.001 |
| rdp3_impactscore ~ Ext | 0.27 | 0.05 | <0.001 |
| rdp3_impactscore ~ WB | -0.50 | 0.07 | <0.001 |
| dp1_impactscore ~ gender | -0.18 | 0.05 | <0.001 |
| dp1_impactscore ~ asian | -0.54 | 0.09 | <0.001 |
| dp1_impactscore ~ black | -0.36 | 0.10 | <0.001 |
| dp1_impactscore ~ mixed | -0.10 | 0.11 | 0.376 |
| dp1_impactscore ~ other | -0.16 | 0.12 | 0.182 |
| dp1_impactscore ~ npd_everfsm | 0.46 | 0.05 | <0.001 |
| dp1_impactscore ~ npd_all_sen | 0.80 | 0.06 | <0.001 |
| dp1_impactscore ~ IDACIScore | 0.38 | 0.16 | <0.05 |
| dp1_impactscore ~ Int | 1.82 | 0.06 | <0.001 |
| dp1_impactscore ~ Ext | 0.72 | 0.04 | <0.001 |
| dp1_impactscore ~ WB | -0.81 | 0.06 | <0.001 |
| dp1_sdq3 \| t1 | 0.02 | 0.05 | 0.692 |
| dp1_sdq3 \| t2 | 1.15 | 0.05 | <0.001 |
| dp1_sdq8 \| t1 | -0.97 | 0.05 | <0.001 |
| dp1_sdq8 \| t2 | 0.11 | 0.05 | <0.05 |
| dp1_sdq13 \| t1 | 0.44 | 0.05 | <0.001 |
| dp1_sdq13 \| t2 | 1.51 | 0.05 | <0.001 |
| dp1_sdq16 \| t1 | -0.72 | 0.05 | <0.001 |
| dp1_sdq16 \| t2 | 0.42 | 0.05 | <0.001 |
| dp1_sdq24 \| t1 | -0.21 | 0.05 | <0.001 |
| dp1_sdq24 \| t2 | 0.85 | 0.05 | <0.001 |
| dp1_sdq5 \| t1 | 0.86 | 0.05 | <0.001 |
| dp1_sdq5 \| t2 | 1.89 | 0.05 | <0.001 |
| dp1_sdq7_reversed \| t1 | 0.58 | 0.05 | <0.001 |
| dp1_sdq7_reversed \| t2 | 2.29 | 0.05 | <0.001 |
| dp1_sdq12 \| t1 | 1.74 | 0.06 | <0.001 |
| dp1_sdq12 \| t2 | 2.71 | 0.06 | <0.001 |
| dp1_sdq18 \| t1 | 1.17 | 0.05 | <0.001 |
| dp1_sdq18 \| t2 | 2.11 | 0.05 | <0.001 |
| dp1_sdq22 \| t1 | 2.10 | 0.07 | <0.001 |
| dp1_sdq22 \| t2 | 2.88 | 0.07 | <0.001 |
| dp1_swemwebs1 \| t1 | -1.82 | 0.05 | <0.001 |
| dp1_swemwebs1 \| t2 | -1.13 | 0.05 | <0.001 |
| dp1_swemwebs1 \| t3 | -0.28 | 0.04 | <0.001 |
| dp1_swemwebs1 \| t4 | 0.65 | 0.05 | <0.001 |
| dp1_swemwebs2 \| t1 | -1.71 | 0.05 | <0.001 |
| dp1_swemwebs2 \| t2 | -0.93 | 0.04 | <0.001 |
| dp1_swemwebs2 \| t3 | -0.01 | 0.04 | 0.852 |
| dp1_swemwebs2 \| t4 | 1.00 | 0.05 | <0.001 |
| dp1_swemwebs3 \| t1 | -1.39 | 0.05 | <0.001 |
| dp1_swemwebs3 \| t2 | -0.53 | 0.05 | <0.001 |
| dp1_swemwebs3 \| t3 | 0.31 | 0.04 | <0.001 |
| dp1_swemwebs3 \| t4 | 1.18 | 0.05 | <0.001 |
| dp1_swemwebs4 \| t1 | -1.84 | 0.05 | <0.001 |
| dp1_swemwebs4 \| t2 | -1.12 | 0.05 | <0.001 |
| dp1_swemwebs4 \| t3 | -0.32 | 0.04 | <0.001 |
| dp1_swemwebs4 \| t4 | 0.55 | 0.05 | <0.001 |
| dp1_swemwebs5 \| t1 | -1.98 | 0.05 | <0.001 |
| dp1_swemwebs5 \| t2 | -1.22 | 0.05 | <0.001 |
| dp1_swemwebs5 \| t3 | -0.36 | 0.05 | <0.001 |
| dp1_swemwebs5 \| t4 | 0.58 | 0.05 | <0.001 |
| dp1_swemwebs6 \| t1 | -2.20 | 0.05 | <0.001 |
| dp1_swemwebs6 \| t2 | -1.54 | 0.05 | <0.001 |
| dp1_swemwebs6 \| t3 | -0.83 | 0.05 | <0.001 |
| dp1_swemwebs6 \| t4 | 0.01 | 0.05 | 0.769 |
| dp1_swemwebs7 \| t1 | -2.10 | 0.05 | <0.001 |
| dp1_swemwebs7 \| t2 | -1.46 | 0.05 | <0.001 |
| dp1_swemwebs7 \| t3 | -0.67 | 0.05 | <0.001 |
| dp1_swemwebs7 \| t4 | 0.22 | 0.05 | <0.001 |
| dp1_sdq3 ~~ dp1_sdq3 | 0.72 | 0.00 | NA |
| dp1_sdq8 ~~ dp1_sdq8 | 0.51 | 0.00 | NA |
| dp1_sdq13 ~~ dp1_sdq13 | 0.28 | 0.00 | NA |
| dp1_sdq16 ~~ dp1_sdq16 | 0.67 | 0.00 | NA |
| dp1_sdq24 ~~ dp1_sdq24 | 0.67 | 0.00 | NA |
| dp1_sdq5 ~~ dp1_sdq5 | 0.41 | 0.00 | NA |
| dp1_sdq7_reversed ~~ dp1_sdq7_reversed | 0.75 | 0.00 | NA |
| dp1_sdq12 ~~ dp1_sdq12 | 0.62 | 0.00 | NA |
| dp1_sdq18 ~~ dp1_sdq18 | 0.55 | 0.00 | NA |
| dp1_sdq22 ~~ dp1_sdq22 | 0.77 | 0.00 | NA |
| dp1_swemwebs1 ~~ dp1_swemwebs1 | 0.79 | 0.00 | NA |
| dp1_swemwebs2 ~~ dp1_swemwebs2 | 0.62 | 0.00 | NA |
| dp1_swemwebs3 ~~ dp1_swemwebs3 | 0.55 | 0.00 | NA |
| dp1_swemwebs4 ~~ dp1_swemwebs4 | 0.52 | 0.00 | NA |
| dp1_swemwebs5 ~~ dp1_swemwebs5 | 0.41 | 0.00 | NA |
| dp1_swemwebs6 ~~ dp1_swemwebs6 | 0.72 | 0.00 | NA |
| dp1_swemwebs7 ~~ dp1_swemwebs7 | 0.55 | 0.00 | NA |
| rdp3_impactscore ~~ rdp3_impactscore | 6.31 | 0.08 | <0.001 |
| dp1_impactscore ~~ dp1_impactscore | 4.52 | 0.06 | <0.001 |
| Int ~~ Int | 0.28 | 0.01 | <0.001 |
| Ext ~~ Ext | 0.59 | 0.01 | <0.001 |
| WB ~~ WB | 0.21 | 0.01 | <0.001 |
| Int ~~ Ext | 0.20 | 0.01 | <0.001 |
| Int ~~ WB | -0.12 | 0.00 | <0.001 |
| Ext ~~ WB | -0.17 | 0.00 | <0.001 |
| gender ~~ gender | 0.25 | 0.00 | NA |
| gender ~~ asian | -0.01 | 0.00 | NA |
| gender ~~ black | 0.00 | 0.00 | NA |
| gender ~~ mixed | 0.00 | 0.00 | NA |
| gender ~~ other | 0.00 | 0.00 | NA |
| gender ~~ npd_everfsm | 0.00 | 0.00 | NA |
| gender ~~ npd_all_sen | 0.02 | 0.00 | NA |
| gender ~~ IDACIScore | 0.00 | 0.00 | NA |
| asian ~~ asian | 0.09 | 0.00 | NA |
| asian ~~ black | -0.01 | 0.00 | NA |
| asian ~~ mixed | 0.00 | 0.00 | NA |
| asian ~~ other | 0.00 | 0.00 | NA |
| asian ~~ npd_everfsm | 0.00 | 0.00 | NA |
| asian ~~ npd_all_sen | 0.00 | 0.00 | NA |
| asian ~~ IDACIScore | 0.00 | 0.00 | NA |
| black ~~ black | 0.06 | 0.00 | NA |
| black ~~ mixed | 0.00 | 0.00 | NA |
| black ~~ other | 0.00 | 0.00 | NA |
| black ~~ npd_everfsm | 0.01 | 0.00 | NA |
| black ~~ npd_all_sen | 0.00 | 0.00 | NA |
| black ~~ IDACIScore | 0.00 | 0.00 | NA |
| mixed ~~ mixed | 0.04 | 0.00 | NA |
| mixed ~~ other | 0.00 | 0.00 | NA |
| mixed ~~ npd_everfsm | 0.01 | 0.00 | NA |
| mixed ~~ npd_all_sen | 0.00 | 0.00 | NA |
| mixed ~~ IDACIScore | 0.00 | 0.00 | NA |
| other ~~ other | 0.03 | 0.00 | NA |
| other ~~ npd_everfsm | 0.00 | 0.00 | NA |
| other ~~ npd_all_sen | 0.00 | 0.00 | NA |
| other ~~ IDACIScore | 0.00 | 0.00 | NA |
| npd_everfsm ~~ npd_everfsm | 0.23 | 0.00 | NA |
| npd_everfsm ~~ npd_all_sen | 0.02 | 0.00 | NA |
| npd_everfsm ~~ IDACIScore | 0.02 | 0.00 | NA |
| npd_all_sen ~~ npd_all_sen | 0.11 | 0.00 | NA |
| npd_all_sen ~~ IDACIScore | 0.00 | 0.00 | NA |
| IDACIScore ~~ IDACIScore | 0.02 | 0.00 | NA |
| dp1_sdq3 ~*~ dp1_sdq3 | 1.00 | 0.00 | NA |
| dp1_sdq8 ~*~ dp1_sdq8 | 1.00 | 0.00 | NA |
| dp1_sdq13 ~*~ dp1_sdq13 | 1.00 | 0.00 | NA |
| dp1_sdq16 ~*~ dp1_sdq16 | 1.00 | 0.00 | NA |
| dp1_sdq24 ~*~ dp1_sdq24 | 1.00 | 0.00 | NA |
| dp1_sdq5 ~*~ dp1_sdq5 | 1.00 | 0.00 | NA |
| dp1_sdq7_reversed ~*~ dp1_sdq7_reversed | 1.00 | 0.00 | NA |
| dp1_sdq12 ~*~ dp1_sdq12 | 1.00 | 0.00 | NA |
| dp1_sdq18 ~*~ dp1_sdq18 | 1.00 | 0.00 | NA |
| dp1_sdq22 ~*~ dp1_sdq22 | 1.00 | 0.00 | NA |
| dp1_swemwebs1 ~*~ dp1_swemwebs1 | 1.00 | 0.00 | NA |
| dp1_swemwebs2 ~*~ dp1_swemwebs2 | 1.00 | 0.00 | NA |
| dp1_swemwebs3 ~*~ dp1_swemwebs3 | 1.00 | 0.00 | NA |
| dp1_swemwebs4 ~*~ dp1_swemwebs4 | 1.00 | 0.00 | NA |
| dp1_swemwebs5 ~*~ dp1_swemwebs5 | 1.00 | 0.00 | NA |
| dp1_swemwebs6 ~*~ dp1_swemwebs6 | 1.00 | 0.00 | NA |
| dp1_swemwebs7 ~*~ dp1_swemwebs7 | 1.00 | 0.00 | NA |
| dp1_sdq3 ~1 | 0.00 | 0.00 | NA |
| dp1_sdq8 ~1 | 0.00 | 0.00 | NA |
| dp1_sdq13 ~1 | 0.00 | 0.00 | NA |
| dp1_sdq16 ~1 | 0.00 | 0.00 | NA |
| dp1_sdq24 ~1 | 0.00 | 0.00 | NA |
| dp1_sdq5 ~1 | 0.00 | 0.00 | NA |
| dp1_sdq7_reversed ~1 | 0.00 | 0.00 | NA |
| dp1_sdq12 ~1 | 0.00 | 0.00 | NA |
| dp1_sdq18 ~1 | 0.00 | 0.00 | NA |
| dp1_sdq22 ~1 | 0.00 | 0.00 | NA |
| dp1_swemwebs1 ~1 | 0.00 | 0.00 | NA |
| dp1_swemwebs2 ~1 | 0.00 | 0.00 | NA |
| dp1_swemwebs3 ~1 | 0.00 | 0.00 | NA |
| dp1_swemwebs4 ~1 | 0.00 | 0.00 | NA |
| dp1_swemwebs5 ~1 | 0.00 | 0.00 | NA |
| dp1_swemwebs6 ~1 | 0.00 | 0.00 | NA |
| dp1_swemwebs7 ~1 | 0.00 | 0.00 | NA |
| rdp3_impactscore ~1 | 2.49 | 0.11 | <0.001 |
| dp1_impactscore ~1 | 0.81 | 0.11 | <0.001 |
| gender ~1 | 1.47 | 0.00 | NA |
| asian ~1 | 0.10 | 0.00 | NA |
| black ~1 | 0.06 | 0.00 | NA |
| mixed ~1 | 0.04 | 0.00 | NA |
| other ~1 | 0.03 | 0.00 | NA |
| npd_everfsm ~1 | 1.37 | 0.00 | NA |
| npd_all_sen ~1 | 1.13 | 0.00 | NA |
| IDACIScore ~1 | 0.25 | 0.00 | NA |
| Int ~1 | 0.00 | 0.00 | NA |
| Ext ~1 | 0.00 | 0.00 | NA |
| WB ~1 | 0.00 | 0.00 | NA |
| direct_Int := a | 0.76 | 0.07 | <0.001 |
| indirect_Int := b*g | 0.41 | 0.02 | <0.001 |
| total_Int := (b*g)+a | 1.17 | 0.07 | <0.001 |
| direct_Ext := c | 0.27 | 0.05 | <0.001 |
| indirect_Ext := d*g | 0.16 | 0.01 | <0.001 |
| total_Ext := (d*g)+c | 0.44 | 0.05 | <0.001 |
| direct_WB := e | -0.50 | 0.07 | <0.001 |
| indirect_WB := f*g | -0.18 | 0.02 | <0.001 |
| total_WB := (f*g)+e | -0.68 | 0.07 | <0.001 |
