# Supplementary material for: Incorporating wellbeing into general factor models: A more complete mental state?
Source: PLoS One. 2025 Nov 17;20(11):e0335657. doi: 10.1371/journal.pone.0335657 (PMC12622774; doi:10.1371/journal.pone.0335657)
Supplement: S5 Table — (DOCX) [file pone.0335657.s005.docx]

**S5 Table. Unadjusted models with specific factors.**

|  | **​Variables** | **Pooled standardized estimate ​** | **Pooled standardized standard error ​** |
| --- | --- | --- | --- |
| **Unadjusted correlated factors INT mediation model** | | | |
| ​​​​Chi-squared <0.01  CFI: 0.907  TLI: 0.894  RMSEA: 0.049  SRMR: 0.060 | Future imp ~ INT | 0.32* | 0.02 |
|  | Future imp  ~ concurrent imp | 0.15* | 0.013 |
|  | concurrent imp ~ INT | 0.65* | 0.006 |
| **Unadjusted correlated factors EXT mediation model** | | | |
| ​  ​Chi-squared < 0.001  CFI: 0.898  TLI: 0.884  RMSEA: 0.052​  SRMR: 0.066  ​ | future imp ~ EXT | 0.34* | 0.017 |
|  | future imp ~ concurrent imp​ | 0.13* | 0.014 |
|  | concurrent imp ~ EXT​ | 0.67* | 0.007 |
| **Unadjusted correlated factors WB mediation model** | | | |
| Chi-squared < 0.001  CFI: 0.886  TLI: 0.871  RMSEA: 0.055  SRMR: 0.069 | Future imp ~ WB | -0.23* | 0.012 |
|  | Future imp  ~ concurrent imp | 0.23* | 0.011 |
|  | concurrent imp ~ WB | -0.55* | 0.006 |
