# Supplementary material for: Incorporating wellbeing into general factor models: A more complete mental state?
Source: PLoS One. 2025 Nov 17;20(11):e0335657. doi: 10.1371/journal.pone.0335657 (PMC12622774; doi:10.1371/journal.pone.0335657)
Supplement: S6 Table — (DOCX) [file pone.0335657.s006.docx]

**S6 Table. Sensitivity Analyses**

S6 Table A. Measurement models estimated with MLR estimator with full-information maximum likelihood.

|  | **Bifactor g_wb_ with method factor** | **Bifactor p** | **Correlated factors mediation model** |
| --- | --- | --- | --- |
| **Degrees of freedom** | 101 | 25 | 116 |
| **Chi-square** | 2593.774 | 933.009 | 4576.374 |
| **Robust CFI** | 0.950 | 0.962 | 0.906 |
| **Robust TLI** | 0.932 | 0.931 | 0.890 |
| **Robust RMSEA** | 0.043 | 0.051 | 0.055 |
| **SRMR** | 0.033 | 0.028 | 0.047 |

S6 Table B. Structural models estimated with MLR estimator with full-information maximum likelihood.

|  | **Bifactor g_wb_ with method factor** | **Bifactor p** | **Correlated factors mediation model** |
| --- | --- | --- | --- |
| **Degrees of freedom** | 263 | 119 | 280 |
| **Chi-square** | 5838.502 | 3709.791 | 7919.649 |
| **Robust CFI** | 0.907 | 0.894 | 0.871 |
| **Robust TLI** | 0.886 | 0.856 | 0.852 |
| **Robust RMSEA** | 0.040 | 0.047 | 0.046 |
| **SRMR** | 0.041 | 0.044 | 0.047 |

S6 Table C. Structural models estimated using WLSMV without multiple imputation

|  | **Bifactor g_wb_ with method factor** | **Bifactor p** | **Correlated factors mediation model** |
| --- | --- | --- | --- |
| **Degrees of freedom** | 263 | 119 | 280 |
| **Chi-square** | 4503.713 | 2863.0 | 6114.26 |
| **CFI** | 0.942 | 0.921 | 0.914 |
| **TLI** | 0.929 | 0.892 | 0.902 |
| **RMSEA** | 0.043 | 0.050 | 0.049 |
| **SRMR** | 0.037 | 0.032 | 0.053 |

S6 Table D. Fit indices of bifactor g_wb_ without method factor mediation model (using WLSMV estimator with multiple imputation)

| **Degrees of freedom** | 264 |
| --- | --- |
| **Chi-square** | 7748.917 |
| **CFI** | 0.934 |
| **TLI** | 0.919 |
| **RMSEA** | 0.043 |
| **SRMR** | 0.043 |
