# Supplementary material for: Incorporating wellbeing into general factor models: A more complete mental state?
Source: PLoS One. 2025 Nov 17;20(11):e0335657. doi: 10.1371/journal.pone.0335657 (PMC12622774; doi:10.1371/journal.pone.0335657)
Supplement: S7 Text — (DOCX) [file pone.0335657.s007.docx]

**S7 Text: Additional analyses**

As per reviewer suggestion, we conducted some additional analyses. We conducted this additional analysis on an archived and fully anonymized version of the original dataset. The original dataset has been destroyed in line with ethical requirements and is no longer available for further analyses. We would like to note that:

- The final quantitative sample sizes in the archived data does vary slightly from the original sample used due to some data being removed in the process of anonymising and preparing the data for public archiving. Specifically, the archived data has n = 15255 rather than n = 15258.
- We were not able to include the following variables in the archived dataset, as they were not part of the original survey but were obtained using a linkage with the National Pupil Database: ethnicity, FSM (free school meals) status, SEN (special educational needs) status and IDACI (area deprivation) score. This specific issue does not affect the internal consistency reliability analysis, as this was conducted on the sample without multiple imputation. However, the other requested analyses below are conducted on multiply imputed data and the results vary very slightly (mostly, at the level of the 2^nd^ or 3^rd^ decimal point) because of the presence of 4 less socio-demographic variables during the imputation.
- Overall, we believe that the results with the archived dataset and the original sample would have been very similar for these additional analyses. We are confident of this, based on the fact that calculated results are very similar for any reproduced analyses (e.g. see Table 1 and Table 1A below; factor loadings for models on archived datasets were very similar to original datasets, only differing at the third decimal point in some instances).

**Internal consistency reliability analysis**

The unidimensional internal consistency reliability for the sample of n = 15255 (using all available data) is as following:

| ***Scale*** | ***Cronbach’s alpha*** |
| --- | --- |
| SWEMWBS | 0.8 (CI: 0.79 – 0.8) |
| SDQ internalizing items | 0.72 (CI: 0.71 – 0.72) |
| SDQ externalizing items | 0.65 (CI: 0.64 – 0.66) |

**Pure unidimensional model**

As per the reviewer’s suggestion, we also fit an additional measurement model which just included a general factor loading on all SDQ and SWEMWBS items with no specific or method factors. This model showed poor fit, as per the predefined fit thresholds. This suggests that a single general factor is not adequate to explain the variance in the data.

Below, we first present the original table with the measurement models, as presented in the mansucript and then an updated table, when we reran the models on the archived dataset, including an additional pure unidimensional model. Note that in all cases in Table 1A, the fit indices remain very similar.

**Table S7 A. Measurement Models, with original dataset (as presented in the manuscript)**

| **Model** | **Chi-squared** | **CFI*** | **TLI *** | **RMSEA*** | **SRMR *** |
| --- | --- | --- | --- | --- | --- |
| Bifactor g_wb_ | p < 0.001 | 0.948 | 0.931 | 0.056 | 0.049 |
| Correlated factors | p < 0.001 | 0.922 | 0.908 | 0.065 | 0.064 |
| Bifactor g_wb_ with method | p < 0.001 | 0.959 | 0.945 | 0.050 | 0.045 |
| Bifactor p | p < 0.001 | 0.971 | 0.949 | 0.054 | 0.042 |
| Bifactor with method factors | p < 0.001 | 0.927 | 0.903 | 0.067 | 0.059 |

g_wb_, general factor with wellbeing; p, general psychopathology factor; CFI, Comparative Fit Index; TLI, Tucker-Lewis Index, RMSEA, Root Mean Square Error of Approximation; SRMR, Standardized Root Mean Square Residual.

*Predefined thresholds: CFI & TLI: Acceptable >0.90, Excellent > 0.95; RMSEA: Acceptable < 0.08, Excellent < 0.06; SRMR <0.08

**Table S7 B. Measurement Models, with archived dataset**

| **Model** | **Chi-squared** | **CFI*** | **TLI *** | **RMSEA*** | **SRMR *** |
| --- | --- | --- | --- | --- | --- |
| Bifactor g_wb_ | p < 0.001 | 0.948 | 0.931 | 0.056 | 0.049 |
| Correlated factors | p < 0.001 | 0.925 | 0.912 | 0.064 | 0.064 |
| Bifactor g_wb_ with method | p < 0.001 | 0.960 | 0.946 | 0.050 | 0.045 |
| Bifactor p | p < 0.001 | 0.970 | 0.947 | 0.055 | 0.043 |
| Bifactor with method factors | p < 0.001 | 0.925 | 0.900 | 0.068 | 0.059 |
| Pure unidimensional | P < 0.001 | 0.758 | 0.723 | 0.113 | 0.116 |

g_wb_, general factor with wellbeing; p, general psychopathology factor; CFI, Comparative Fit Index; TLI, Tucker-Lewis Index, RMSEA, Root Mean Square Error of Approximation; SRMR, Standardized Root Mean Square Residual.

*Predefined thresholds: CFI & TLI: Acceptable >0.90, Excellent > 0.95; RMSEA: Acceptable < 0.08, Excellent < 0.06; SRMR <0.08
