# Supplementary material for: Incorporating wellbeing into general factor models: A more complete mental state?
Source: PLoS One. 2025 Nov 17;20(11):e0335657. doi: 10.1371/journal.pone.0335657 (PMC12622774; doi:10.1371/journal.pone.0335657)
Supplement: Attachment — Submitted filename: Response to Reviewers.docx [file pone.0335657.s009.docx]

**RESPONSE TO REVIEWER COMMENTS**

**Reviewer #1**

***Thank you for the opportunity to review the article “Incorporating wellbeing into general factor models: a more complete mental state?”. The paper is thorough, well presented, and addresses an interesting topic within the literature, namely whether global mental health status might be modelled more accurately through the inclusion of wellbeing alongside psychopathology. The very large sample size, recruited across schools in six different regions of the UK, suggests sufficient statistical power to test the proposed measurement and structural models.***

The authors would like to thank the reviewer for their positive feedback as well as their constructive comments for improvement.

***The method for dealing with missing data was convincing. However, as a small detail, it was unclear to me from the text how the models were fit using the multiple imputed datasets. Was this using the lavaan.mi function? I think readers would benefit if this final part of the imputation process (e.g. pooling) was also explained.***

Thank you to the reviewer for pointing this out. This has now been added to the manuscript (Line 305 - 310).

***It would be of interest for readers to know the unidimensional internal consistency reliability (eg., ω or α) of the measures, with confidence intervals, in the study sample.***

We conducted this as well as another additional analyses (fitting a unidimensional model) on an archived and fully anonymized version of the original dataset (<http://doi.org/10.5255/UKDA-SN-9150-1>). The original, identifiable, dataset has been destroyed due to ethical requirements. While the archived, fully anonymised version is largely similar to the original dataset, we would like to note that:

- The final sample size in the archived data is n = 15255 rather than n = 15258, reflecting minor data removal during anonymisation and preparation for public archiving.
- Several variables originally obtained through linkage with the National Pupil Database (ethnicity, free school meals (FSM) status, special educational needs (SEN) status, and IDACI (area deprivation) score) are not available in the archived dataset.

These differences do not affect the internal consistency reliability analysis, as this was conducted on the sample without multiple imputation, and the results are expected to be identical to those that would have been obtained with the original dataset. However, the other requested analyses below are conducted on multiply imputed data and the results are expected to vary very slightly from the original analyses (mostly, at the level of the 2^nd^ or 3^rd^ decimal point) because of the presence of 4 less socio-demographic variables during the imputation.

These additional analyses are reported in the Supplemental Material (S7: Additional analyses), with a detailed explanation.

***The paper tested several competing measurement models that included indicators of psychopathology and wellbeing. A “bifactor-p” model without wellbeing items was tested as a comparison. The last model tested was labelled as “Unidimensional with method”, which I found misleading, because this is still a bifactor model (which could be contrasted to, for example, a unidimensional model with method factors modelled through correlated residuals). I would consider an alternative label.***

We agree with the reviewer on this point. We have now updated the name to “bifactor with method factors” in the manuscript.

***Although the model was argued not be viable on the basis of the pattern of loadings with the method specific factors, I think readers would still be interested to know the loadings on the general factor.***

We have now updated the manuscript with the relevant details (Line 520 - 521). We also note that all the factor loadings (for both the general and method factors) are also available in S3 Table E.

***Additionally, for comparisons sake, I think readers could be interested in the fit of a pure unidimensional model (no specific factors).***

We specified the pure unidimensional model on the archived dataset, as reported above. The pure unidimensional did not show good fit, as has been the case in previous work (Patalay et al., 2015; Castellanos-Ryan et al., 2016).

We have reported this in the main manuscript (Line 530 - 538), specifying that it is on an archived dataset with further information about comparisons between original and archived datasets in S7: Additional analyses.

***The text refers sometimes to “Model 1” and “Model 2” etc. and I found it difficult to match these labels to the model structures. You could consider putting “Model 1: Bifactor gwb” etc in Table 1 to aid readers.***

We have now added the numbered labels to Table 1. We have also updated the text (e.g. Line 539) to ensure clarity while referring to models by their numbers.

***In relation to the bifactor models, it could be informative to calculate and report bifactor indices such as ωH and ECV. These would offer deeper insights into the uni- vs. multi-dimensionality of the general factors, and by extension issues such as the feasibility of calculating and interpreting total scores across items.***

We have calculated omega hierarchical and explained common variance for the bifactor models fit on the original dataset. For omega hierarchical, we used a custom function that substantively replicated *compRelSEM*’s (semTools) workflow to calculate this. Since our models included negative loadings of SWEMWEBS items on the general factor, directly using *compRelSEM* gave incorrect results for the reliability of the general factor, hence we had to use a custom function to replicate compRelSEM. For explained common variance, we used a custom function based on the BifactorIndices package, applied to pooled standardized factor loadings. Both pipelines used are available on OSF.

***As a small detail, the specific factors for the bifactor gwb with method factor model are referred to as “gwb-free” to appropriately communicate the idea that specific factors explain variance after accounting for the general factor. In terms of interpretation, it is important to communicate that in this model the internalizing and externalizing specific factors are not only ‘gwb-free’, but also ‘method-free’ because they also load on the method factor.***

We thank the reviewer for pointing this out. We have updated the manuscript to refer to these specific factors as gwb-free and method-free.

***After testing the competing measurement models, the authors used SEM to test a series of partial mediation models, each time using a different measurement model (correlated factors model, bifactor p model, and the preferred bifactor gwb model). The mediation model with the preferred bifactor gwb model had the best fit. When reporting the results from this model, the authors also present in the text the associations between the gwb-free specific factors, highlighting as a particular point of interest the positive association between the gwb-free Wellbeing specific factor and concurrent and future impairment. While these results must be presented (as they are in Table 3), I am uncertain whether it is necessary to elaborate them in the text given that it is often very difficult to interpret the meaning of specific factors after factoring out the general factor. As the authors rightly elaborate on in the discussion, “the gwb-free wellbeing specific factor is more difficult to interpret”, meaning ultimately it may well only represent a nuisance effect.***

We had initially described these in the text for transparency rather than substantial interpretation. We have now reduced this elaboration both in the Results and Discussion section, especially for the wellbeing factor, while retaining some discussion about the Int and Ext factors, as they seem relatively more reliable.

***The shared underlying construct (gwb) between wellbeing and psychopathology appears to be a generalized negative mental state”. Firstly, as wellbeing items negatively loaded on the general factor I feel it is misleading to refer to the construct as ‘wellbeing’ in this sentence. I suggest using terminology that better captures this modelled relationship, such as (lack of) wellbeing. I note there are many instances where this is done already in the manuscript, such as in the discussion, but it must be done consistently.***

We have now carefully read through the manuscript and edited this for consistency throughout.

***Second, the discussion lacks consideration of what the general factor might represent other than a “generalized negative mental state”. It could be interesting to explore what this general factor may capture in terms of more stable higher-order, tendencies for illbeing and psychopathology. As an example, one could refer to Cloninger’s psychobiological model of personality for this purpose.***

We have been deliberately tempered in our substantive interpretations of the general factor, in light of recent critiques of etiological interpretations of general factor modelling. However, in agreement with the reviewer’s point, we have now balanced this and added a section to the manuscript (Line 700-712) reflecting on what our g_wb_ factor might represent.

**Editor’s comments**

***This study has a large sample size, employs innovative methods, and presents a well-structured introduction. The data provided in the supplementary materials are also robust.***

***However, I recommend standardizing the format of the p-values to avoid using scientific notation.***

We have updated this in the supplementary materials to reflect p-value thresholds (<0.05, <0.01 and <0.001) instead of scientific notation.

***In addition, I suggest expanding the discussion section to include more practical implications of the methods used, which would help other researchers better understand and apply them.***

We have now added implications of using structural equation modelling to the manuscript in Lines 763 – 774, especially how they can be differentiated from sum score approaches. Further, Line 862-872 also contains practical implications of using structural equation modelling when measuring mental health status.

**Additional Requirements**

***1. Please ensure that your manuscript meets PLOS ONE's style requirements, including those for file naming. The PLOS ONE style templates can be found at***

***https://journals.plos.org/plosone/s/file?id=wjVg/PLOSOne_formatting_sample_main_body.pdf and***

[***https://journals.plos.org/plosone/s/file?id=ba62/PLOSOne_formatting_sample_title_authors_affiliations.pdf***](https://journals.plos.org/plosone/s/file?id=ba62/PLOSOne_formatting_sample_title_authors_affiliations.pdf)

We have gone through the manuscript and corrected all stylistic issues we could find. Please let us know if there is anything we have missed.

***2. We note that you have indicated that there are restrictions to data sharing for this study. PLOS only allows data to be available upon request if there are legal or ethical restrictions on sharing data publicly. For more information on unacceptable data access restrictions, please see http://journals.plos.org/plosone/s/data-availability#loc-unacceptable-data-access-restrictions.***

***Before we proceed with your manuscript, please address the following prompts:***

***a) If there are ethical or legal restrictions on sharing a de-identified data set, please explain them in detail (e.g., data contain potentially identifying or sensitive patient information, data are owned by a third-party organization, etc.) and who has imposed them (e.g., a Research Ethics Committee or Institutional Review Board, etc.). Please also provide contact information for a data access committee, ethics committee, or other institutional body to which data requests may be sent.***

***b) If there are no restrictions, please upload the minimal anonymized data set necessary to replicate your study findings to a stable, public repository and provide us with the relevant URLs, DOIs, or accession numbers. For a list of recommended repositories, please see***

***https://journals.plos.org/plosone/s/recommended-repositories. You also have the option of uploading the data as Supporting Information files, but we would recommend depositing data directly to a data repository if possible.***

***We will update your Data Availability statement on your behalf to reflect the information you provide.***

We would like to clarify that an anonymized version of the dataset used in this study is available publicly at: <http://doi.org/10.5255/UKDA-SN-9150-1>. However, due to the process of anonymisation as well as the ethical restrictions on data storage, the archived dataset does not include four variables (IDACI score, ethnicity, Free School Meals (FSM) status, and Special Educational Needs (SEN) status) and contains three fewer participants than the original study sample used for the analyses in this paper. These four variables were not part of the original survey; they were derived through linkage with the National Pupil Database (NPD). Under current security standards, NPD-linked data can only be archived in specific secure platforms (such as the ONS Secure Research Service) and therefore could not be deposited in the UK Data Service. Therefore, the survey results without the NPD-linked variables were archived in the UK Data Service. Nevertheless, we believe that the substantive conclusions of this study should be able to be replicated by interested readers using this dataset and our methods, as we have demonstrated in our revised manuscript as well.

***3. Please review your reference list to ensure that it is complete and correct. If you have cited papers that have been retracted, please include the rationale for doing so in the manuscript text, or remove these references and replace them with relevant current references. Any changes to the reference list should be mentioned in the rebuttal letter that accompanies your revised manuscript. If you need to cite a retracted article, indicate the article’s retracted status in the References list and also include a citation and full reference for the retraction notice.***

We have added two references which were missing from the reference list.

References

1. Castellanos-Ryan, Natalie, Frederic N. Brière, Maeve O’Leary-Barrett, et al. ‘The Structure of Psychopathology in Adolescence and Its Common Personality and Cognitive Correlates.’ *Journal of Abnormal Psychology* 125, no. 8 (2016): 1039–52. <https://doi.org/10.1037/abn0000193>.
2. Patalay, Praveetha, Peter Fonagy, Jessica Deighton, Jay Belsky, Panos Vostanis, and Miranda Wolpert. ‘A General Psychopathology Factor in Early Adolescence’. *British Journal of Psychiatry* 207, no. 1 (2015): 15–22. <https://doi.org/10.1192/bjp.bp.114.149591>.
